# Evidence for a dominantly reducing Archaean ambient mantle from two redox proxies, and low oxygen fugacity of deeply subducted oceanic crust

**DOI:** 10.1038/s41598-019-55743-1

**Published:** 2019-12-27

**Authors:** Sonja Aulbach, Alan B. Woodland, Richard A. Stern, Prokopiy Vasilyev, Larry M. Heaman, K. S. Viljoen

**Affiliations:** 1 Institute for Geosciences, Goethe-Universität, Altenhöferallee 1, Frankfurt am Main, Germany; 2 Canadian Centre for Isotopic Microanalysis, Department of Earth and Atmospheric Sciences, University of Alberta, Edmonton AB, T6G 2E3 Canada; 3 John de Laeter Centre, Curtin University, Perth, Western Australia Australia; 4 Department of Earth and Atmospheric Sciences, University of Alberta, Edmonton AB, T6G 2E3 Canada; 5 Department of Geology, University of Johannesburg, PO Box 524, Auckland Park 2006 Johannesburg, South Africa

**Keywords:** Petrology, Geochemistry

## Abstract

Oxygen fugacity (ƒO_2_) is an intensive variable implicated in a range of processes that have shaped the Earth system, but there is controversy on the timing and rate of oxidation of the uppermost convecting mantle to its present ƒO_2_ around the fayalite-magnetite-quartz oxygen buffer. Here, we report Fe^3+^/ΣFe and ƒO_2_ for ancient eclogite xenoliths with oceanic crustal protoliths that sampled the coeval ambient convecting mantle. Using new and published data, we demonstrate that in these eclogites, two redox proxies, V/Sc and Fe^3+^/ΣFe, behave sympathetically, despite different responses of their protoliths to differentiation and post-formation degassing, seawater alteration, devolatilisation and partial melting, testifying to an unexpected robustness of Fe^3+^/ΣFe. Therefore, these processes, while causing significant scatter, did not completely obliterate the underlying convecting mantle signal. Considering only unmetasomatised samples with non-cumulate and little-differentiated protoliths, V/Sc and Fe^3+^/ΣFe in two Archaean eclogite suites are significantly lower than those of modern mid-ocean ridge basalts (MORB), while a third suite has ratios similar to modern MORB, indicating redox heterogeneity. Another major finding is the predominantly low though variable estimated ƒO_2_ of eclogite at mantle depths, which does not permit stabilisation of CO_2_-dominated fluids or pure carbonatite melts. Conversely, low-ƒO_2_ eclogite may have caused efficient reduction of CO_2_ in fluids and melts generated in other portions of ancient subducting slabs, consistent with eclogitic diamond formation ages, the disproportionate frequency of eclogitic diamonds relative to the subordinate abundance of eclogite in the mantle lithosphere and the general absence of carbonate in mantle eclogite. This indicates carbon recycling at least to depths of diamond stability and may have represented a significant pathway for carbon ingassing through time.

## Introduction

The melting relations of the convecting mantle and the behaviour of elements during partial melting vary as a function of pressure, temperature and redox state^1–6^. At the time of core formation, presuming that the silicate mantle was in equilibrium with metal, the uppermost convecting mantle had ƒO_2_ relative to the Fayalite-Magnetite-Quartz oxygen buffer (FMQ, reported as ∆logƒO_2_ (FMQ)), of about −4.5, whereas presently values around FMQ are recorded^4–6^, but there is disagreement on the timing and rate of this oxidation. The behaviour of multi-valent elements (e.g. Fe, Eu, V), which depends on their redox state^7^, in basalts has been used to infer that ƒO_2_ in the convecting mantle has been similar to the present day from ca. 3.9 Ga^5^. In contrast, there is recent evidence for a subtle but significant terrestrial mantle redox evolution between 3.5 and 1.9 Ga based on the behaviour of V^8–10^. Moreover, recent studies reveal that garnet in mantle eclogites has low Fe^3+^/ΣFe, typically ≪ 0.10^11–13^, which may be related either to Fe^3+^ loss during partial melting in subduction zones or to an intrinsically more reducing convecting mantle source to the eclogites’ mafic protoliths^8,12,13^.

Here, we investigate eclogite and pyroxenite xenoliths derived from cratonic (>2.5 Ga) mantle lithosphere that have unambiguous signatures of a Palaeoproterozoic to Mesoarchaean spreading-ridge origin^14^, using new data from three localities (Orapa, Koidu and Diavik; Supplementary Dataset 1) and published geochemical and isotopic analyses. We use these data to extract information on the physical state of the ambient convecting mantle, analogous to how modern MORB samples are used^5,6^. We simultaneously apply two redox proxies to five eclogite suites: (1) The ratio of Fe^3+^ to Fe^2+^ in basalts is controlled by oxygen content, such that the average Fe^3+^/ΣFe can be used to obtain their redox state and infer that of their mantle source^6^. (2) The V/Sc redox proxy is based on V becoming more incompatible with increasing valence state as a function of ƒO_2_, whereas the partitioning of Sc is independent of ƒO_2_^5,7^. Thus, the bulk peridotite-basalt distribution coefficient for V changes by nearly two orders of magnitude for a change in oxygen fugacity between FMQ and FMQ-4 ^7^. In this study, a range of major and trace elements in reconstructed bulk rocks as well as δ^18^O in garnet are employed to decipher the processes that have affected these samples from their formation in ancient spreading-ridges to exhumation via kimberlite magmatism. Oxygen fugacity has been suggested to decrease with pressure in eclogite at constant Fe^3+^/ΣFe based on thermodynamic consideration^13^, and is further expected to vary strongly in the subduction environment due to the juxtaposition of rocks with highly variable redox states^15^. Thus, we use Fe^3+^/ΣFe in garnet to estimate ƒO_2_ using one of the recently formulated Fe-based oxybarometers suitable for eclogites^16^ (Methods), which has implications for the effects of deeply recycled ancient ocean floor on processes in the mantle.

## Samples and Eclogite Petrogenesis

The study utilises new mineral Fe^3+^/ΣFe acquired by Mössbauer spectroscopy and δ^18^O data acquired by secondary ion mass spectrometry (Methods) for kimberlite-borne eclogite and pyroxenite xenoliths from Orapa (Zimbabwe craton; n = 17), Koidu (West African craton; n = 16) and Diavik (central Slave craton; n = 5). These eclogites have been interpreted as subducted oceanic crust that formed by partial melting of ca. 3.0 Ga, 2.7 Ga and 2.0 Ga convecting mantle sources, respectively (Supplementary Text). Their low-pressure origin as basaltic to picritic oceanic crust is evidenced, inter alia, by the presence of Eu anomalies (Eu/Eu* = chondrite-normalised Eu/(Sm*Gd)^0.5), which anti-correlate with total heavy rare earth element contents (ΣHREE) contents requiring the participation of plagioclase in their petrogenesis, and by non-mantle δ^18^O requiring low-temperature seawater alteration^14^. This igneous protolith was subsequently subducted, metamorphosed and in part overprinted during mantle metasomatism (Supplementary Text). The major and trace element compositions of eclogites reveal them to have variably differentiated protoliths encompassing plagioclase-rich cumulates (referred to as gabbroic eclogites with high Eu/Eu*, low ΣHREE) and residual melts (low Eu/Eu*, high ΣHREE), as also suggested by their major-element relationships^14^. High-Mg and high-Ca eclogites represent protoliths having experienced low and advanced degrees of differentiation, respectively, whereas low-Mg eclogites are also more differentiated or may require more FeO-rich sources^14^. The eclogites were subsequently variably affected by seawater alteration (as gauged by δ^18^O, the permil deviation from the VSMOW standard), partial melt loss and metasomatism (as gauged by NMORB-normalised Ce/Yb < 1 and >1, respectively; normalisation indicated by subscript NMORB)^14^. These new data are combined with published studies on mantle eclogites and pyroxenites from Voyageur in the northern Slave craton, which are coeval with their ca. 2 Ga central Slave counterparts^11^, as well as from the Lace kimberlite in the Kaapvaal craton with ca. 3 Ga old protoliths^12^.

## Results

### Oxygen isotopes in garnet

Garnet in eclogite and pyroxenite xenoliths from Orapa (Zimbabwe craton) has δ^18^O ranging from +5.09 to +6.71‰, with metasomatised samples having lower average values (+5.47‰) than non-metasomatised ones (+5.87‰). Values higher than the canonical mantle range (+5.1 to +5.9‰)^17^, suggestive of low-temperature seawater alteration, are observed for four of 16 samples. A smaller range of oxygen isotope ratios is measured for garnet in samples from Koidu, West African craton (+5.09 to +6.18‰) and Diavik, central Slave craton (+5.15 to +6.03‰).

### Fe^3+^/ΣFe in clinopyroxene and garnet

Fe^3+^/ΣFe was measured in garnet from Orapa, Koidu and Diavik, and cpx was additionally measured for samples from Orapa. In cpx, Fe^3+^/ΣFe ranges from 0.10 to 0.32 and is on average higher in metasomatised samples (0.21; n = 8) than in unmetasomatised ones (0.17; n = 9). In coexisting garnet, Fe^3+^/ΣFe ranges from 0.01 to 0.07, with only two of 16 samples having ratios > 0.03 (median 0.02). The lowest value is obtained for garnet in an eclogite with strong cumulate (“gabbroic”) character indicative of a plagioclase-rich protolith (garnet Eu/Eu* = 1.6), although other gabbroic eclogites have higher garnet Fe^3+^/ΣFe (Supplementary Dataset 1). Garnet in samples from Koidu has higher median Fe^3+^/ΣFe (0.04), while that in samples from Diavik shows intermediate values (0.02 to 0.05, median 0.03; n = 5).

### Fe^3+^/ΣFe in reconstructed bulk rocks

Crystal chemical and temperature effects on the Fe^3+^ distribution between garnet and cpx (Supplementary Text) imply that garnet Fe^3+^/ΣFe is not representative of the bulk rock. Eclogite and pyroxenite cpx consistently has higher Fe^3+^/ΣFe than garnet though lower total Fe contents (~20% of the total Fe for samples considered in this study), such that garnet controls bulk Fe^3+^/ΣFe (accessory rutile typically contains a few wt% Fe at most), consistent with experiments^13^. Bulk rock Fe^3+^/ΣFe reconstructed from measured mineral Fe^3+^/ΣFe in Orapa samples ranges from 0.03 ± 0.01 to 0.12 ± 0.01, with values ≤ 0.07 for 14 of 16 samples. For the other sample suites, bulk rock Fe^3+^/ΣFe was reconstructed from measured garnet Fe^3+^/ΣFe and calculated cpx Fe^3+^/ΣFe. The latter was obtained from garnet Fe^3+^/ΣFe and temperatures, based on the temperature-dependent distribution of Fe^3+^/ΣFe between garnet and cpx (Methods). Bulk rock Fe^3+^/ΣFe in eclogites and pyroxenites from Koidu and Diavik range from 0.04 ± 0.03 to 0.16 ± 0.14 and 0.05 ± 0.02 to 0.15 ± 0.11, respectively. Bulk rock reconstruction was also applied to eclogites and pyroxenites from Voyageur (northern Slave craton) and from Lace (Kaapvaal craton) using published garnet Fe^3+^/ΣFe and temperatures that were recalculated for consistency (Supplementary Dataset 1).

### Fe-based oxygen fugacity

Calculated ∆logƒO_2_ (oxybarometer of ref. ^16^) for the Orapa suite span a large range, from FMQ-4.9 + 1.5/ −0.8 to FMQ-1.23 + 0.26/ −0.23. Diavik eclogites show a similar range at lower values, from FMQ-5.5 + 1.5/ −0.8 to FMQ-2.9 + 0.5/ −0.4 (Supplementary Dataset 1), while for Koidu ∆logƒO_2_ is not as variable (FMQ-3.5 + 0.3/ −0.4 to FMQ-1.67 + 0.26/ −0.31). The ∆logƒO_2_ of these eclogites, which form a subordinate lithology in the cratonic lithospheric mantle, is not imposed by the dominant peridotite, as illustrated in Supplementary Fig. 1.

## Discussion

### Effects of post-formation processes on Fe^3+^/ΣFe and V/Sc

Several processes occur between generation of the eclogites’ crustal protoliths in palaeo-spreading ridges and their exhumation via kimberlite magmatism that may affect the proxies used to infer the redox state of their mantle source. These are: Degassing on the seafloor, seawater alteration between the ridge and the trench, partial melt loss during metamorphism and metasomatism due to interaction with fluids and melts during their residence in the cratonic lithosphere.

#### Degassing

Depending on their pressure of emplacement and the nature of the volatile species, degassing of basalts can increase or decrease the Fe^3+^/ΣFe and hence redox state inferred for the magma^17^. Recent work finds no evidence that degassing or interaction with polyvalent gas species, such as S, has affected Fe^3+^/ΣFe in modern MORBs, nor that ƒO_2_ is externally buffered^6^, and we suggest that this also applies to magma emplacement in palaeo-ridges. For degassing to be important, differences in process between the Archean and today would be required. However, even if degassing had affected Fe^3+^/ΣFe, such changes in valence state do not change elemental redox proxies, such as V/Sc, the ratio of which in the undifferentiated magma is set at source (the effects of differentiation are addressed in a later paragraph).

#### Seawater alteration

**Figure 1:**
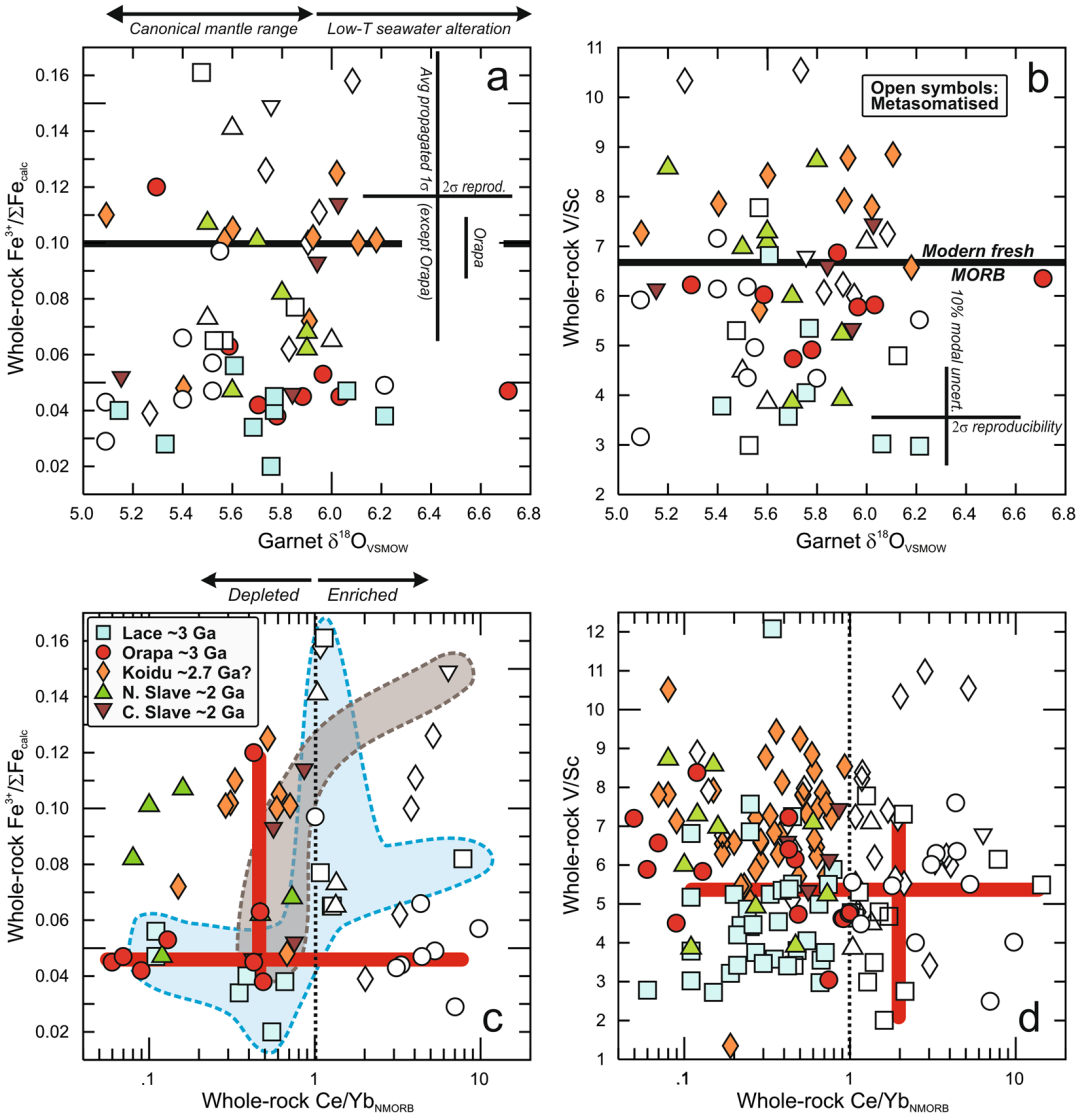
Effects of seawater alteration, metasomatism and melt loss on redox proxies in mantle eclogite. (**a**) Fe^3+^/ΣFe and (**b**) V/Sc in reconstructed whole rocks as a function of δ^18^O (denotes permil deviation from SMOW standard) in garnet as a proxy for seawater alteration^19^. Uncertainties on Fe^3+^/ΣFe are propagated from those on cpx and garnet Fe^3+^/ΣFe, assuming a total 10% uncertainty on the modal proportions, weighted by the proportion of Fe contributed to the bulk rock. Differences in uncertainty between Orapa and other eclogite suites derive from cpx Fe^3+^/ΣFe being measured in the former and calculated in the latter. Uncertainty on V/Sc reflects that resulting from 10% uncertainty on the modal proportions. Canonical mantle range of δ^18^O from^18^; V/Sc and Fe^3+^/ΣFe of modern fresh MORB from^8^ and^25^, respectively. (**c**) Fe^3+^/ΣFe and (**d**) V/Sc as a function of NMORB-normalised (denoted with subscript NMORB) Ce/Yb in reconstructed whole rocks, as a proxy for melt loss from eclogite (< 1) and metasomatism/enrichment (> 1) (NMORB of^37^). Only in the suites from Lace (blue field) and Diavik (light brown field) do metasomatised samples have higher Fe^3+^/ΣFe than unmetasomatised ones. There is no discernible effect of melt depletion on Fe^3+^/ΣFe or V/Sc, which varies little across a wide range of Ce/Yb_N_ for Orapa and Lace eclogites, respectively (horizontal red bars); conversely, these eclogites show a wide range of Fe^3+^/ΣFe at similar degree of melt depletion (vertical red bars).

Unlike fresh MORB, recycled equivalents have experienced variable degrees of seawater alteration, causing deviation of oxygen isotope compositions from the mantle range^18^. We assess this using δ^18^O in garnet, which has been shown to be a reliable proxy for seawater alteration in mantle eclogites^19^. Figure 1a shows that the Fe^3+^/ΣFe of reconstructed bulk rocks is independent of garnet δ^18^O. This result is not unexpected in light of recent evidence for near-constant and low Fe^3+^/ΣFe in seawater-altered oceanic crust before Neoproterozoic oxygenation of oceanic bottom waters occurred^20^. Similarly, V/Sc in reconstructed bulk rocks is independent of evidence for seafloor weathering (Fig. 1b), consistent with the generally fluid-immobile behaviour of V and Sc^5^.

#### Partial melt loss

Melt extraction from eclogite has been linked to the generation of tonalite-trondhjemite-granodiorite magmas forming Archaean continental crust^21^. The effect of partial melt loss from eclogite, presumably during subduction and metamorphism, is assessed using NMORB-normalised Ce/Yb (denoted with subscript NMORB), which decreases to values < 1 as a function of melt fraction extracted (Supplementary Text). This shows that while a large range of Fe^3+^/ΣFe is observed over similar Ce/Yb_NMORB_ (Fig. 1c, see vertical red bars), Fe^3+^/ΣFe varies little as a function of Ce/Yb_NMORB_ (Fig. 1c, see horizontal red bars, using Orapa as an example). This may be explained by retention in residual cpx where Fe^3+^ is less incompatible than Fe^2+^ ^7^. Likewise, there is no clear indication for dependence of V/Sc on Ce/Yb_NMORB_ (Fig. 1d). Thus, samples from all suites also show a wide range of V/Sc at similar Ce/Yb_NMORB_ and similar V/Sc at a range of Ce/Yb_NMORB_ (Fig. 1d, see vertical and horizontal red bars, respectively, using Lace as an example). The immobile behaviour of V during melt loss is consistent with low ƒO_2_ and V compatibility, whereas partial melting at higher ƒO_2_ has been modelled to lead to incompatible behaviour and loss from eclogite during metamorphism, as exhibited by some Proterozoic orogenic eclogite suites^8^.

#### Mantle metasomatism

Any melt formed within or below cratons and affecting the mantle eclogite reservoir at the depth where it resides would most likely leave a garnet-bearing residue and have a small volume because the thickness of cratonic lithospheres leaves little room for decompression melting. As a corollary, mantle metasomatism typically involves LREE-enrichment which is proxied by NMORB-normalised Ce/Yb > 1 (ref. ^14^). Typically oxidising metasomatism is expected to raise Fe^3+^/ΣFe^4^ and can decrease V concentrations in metasomatised rocks because of the higher valence state and lower associated distribution coefficients^22^. Indeed, strongly metasomatised, phlogopite-bearing eclogites from Kimberley in the Kaapvaal craton^23^ have low but variable V/Sc (3.9 ± 2.1). Thus, mantle metasomatism entails contrasting behaviour of the two redox proxies. There are some hints in the data for a link of metasomatism and an increase in Fe^3+^/ΣFe, as metasomatised eclogites from Lace and Diavik, but not from other suites, have higher bulk-rock Fe^3+^/ΣFe than unmetasomatised varieties (coloured fields in Fig. 1c). Conversely, a link between metasomatism and V/Sc is not evident (Fig. 1d).

### Retention of primary Fe^3+^/ΣFe in mantle eclogite xenoliths and sympathetic behaviour with V-based redox sensors

**Figure 2:**
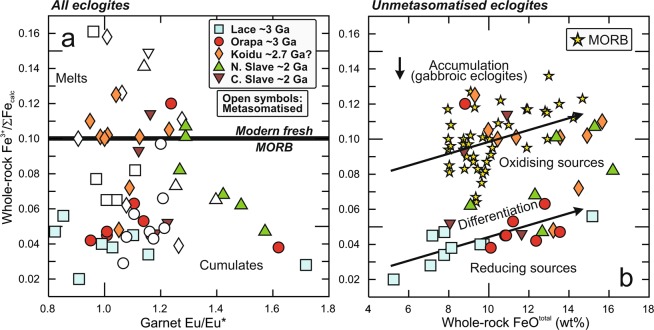
Effects of differentiation (oceanic crustal protolith). Fe^3+^/ΣFe in reconstructed whole rocks as a function of (**a**) Eu/Eu* (chondrite-normalised Eu/(Sm*Gd)^0.5) in garnet, as a proxy for plagioclase accumulation and fractionation during protolith formation, and (**b**) FeO content in reconstructed whole rock mantle eclogite. In each eclogite suite, the lowest or one of the lowest Fe^3+^/ΣFe is observed for samples with a strong cumulate signature (Eu/Eu*≫1). Average modern fresh MORB from^25^. The trend to increasing Fe^3+^/ΣFe with increasing FeO in unmetasomatised eclogites from Lace and Orapa could be related to differentiation in the protoliths, whereas low Fe^3+^/ΣFe in gabbroic eclogites may be due to accumulation; a similar trend is observed in modern fresh MORB (yellow stars)^25^.

**Figure 3:**
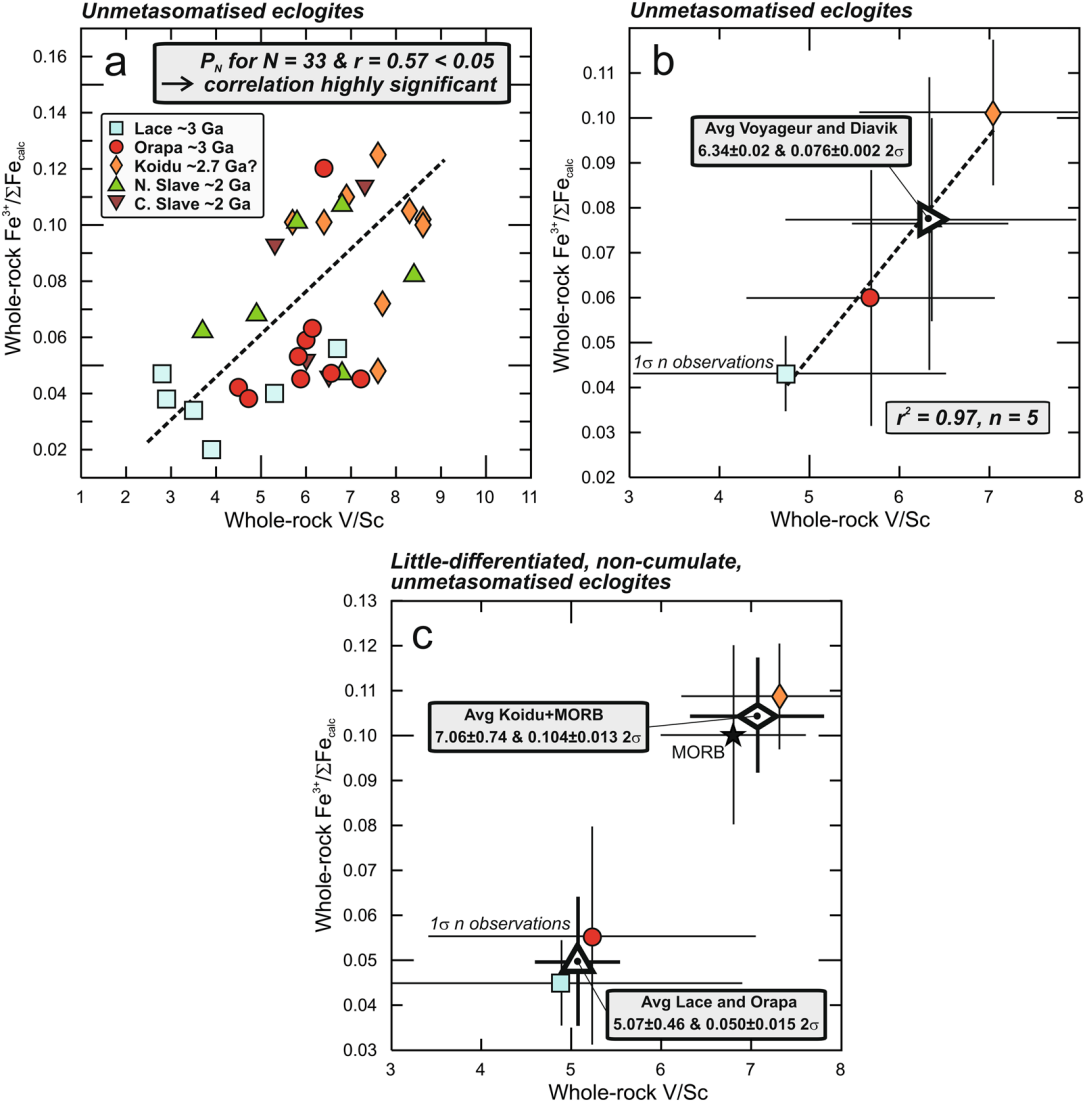
Sympathetic behaviour of Fe^3+^/ΣFe and V/Sc. Reconstructed whole rock mantle eclogite showing (**a**) all individual non-metasomatised samples, (**b**) averages for non-metasomatised samples per locality and (**c**) averages only for non-metasomatised, non-cumulate and little-differentiated samples (that is, excluding high-Ca and gabbroic eclogites^14^). Although the correlation coefficient in (**a**) is seemingly low (r^2^ = 0.32), there is a probability < 0.5% that the two variables are uncorrelated for the number of data points in the regression (n = 33), and the correlation is highly significant^38^. Proterozoic eclogite suites from the northern and central Slave craton have a strong cumulate character, implying that their Fe^3+^/ΣFe, and their V/Sc for accumulation under oxidising conditions, are lower than for eclogites with melt-like protoliths, and they are therefore not considered in (**c**). In (**b**,**c**) averages for V/Sc encompass all available values (references in Supplementary Dataset) and are not limited to samples for which Fe^3+^/ΣFe has been determined; error bars for individual suites are 1 standard deviation for *n* averaged values (Methods).

Several observations suggest that Fe^3+^/ΣFe in unmetasomatised mantle eclogites retains a record of igneous differentiation on the ocean floor, hence inheritance from their protoliths: (1) For all eclogite suites, the lowest or one of the lowest Fe^3+^/ΣFe is associated with high Eu/Eu* in reconstructed bulk rocks (Fig. 2a), which corresponds to the expected relationship if the more incompatible Fe^3+^ (ref. ^6^) is excluded from plagioclase-rich cumulates characterised by Eu/Eu* > 1. (2) Eclogites in unmetasomatised Lace and Orapa samples show generally increasing Fe^3+^/ΣFe with increasing FeO, which is interpreted as a differentiation trend also recognisable in modern MORB (Fig. 2b). (3) A positive correlation between Fe^3+^/ΣFe and V/Sc in unmetasomatised samples is evident (Fig. 3a). As a result of the differential response of Fe^3+^/ΣFe and V/Sc to differentiation, degassing, seawater alteration, partial melt extraction and metasomatism, as detailed above, the variation within each suite composed of eclogites each representing the sum of multiple processes is large. Despite this, the average values obtained per suite clearly show sympathetic behaviour (r^2^ = 0.97; Fig. 3b), which is interpreted to reflect inheritance from the protolith and implies an unexpected robustness of Fe^3+^/ΣFe.

With respect to Fe^3+^/ΣFe, the two Proterozoic eclogite suites are dominated by samples with cumulate protoliths (gabbroic eclogites), with the consequence that these eclogites have inherited lower Fe^3+^/ΣFe from their protoliths than hypothetical complementary residual melts. At the same time, more incompatible behaviour of V under oxidising conditions^7^ implies stronger exclusion from accumulating minerals^5^ and low V/Sc compared to melts. This diminishes the contrast of Proterozoic gabbroic eclogites with their Archaean counterparts, which are dominated by non-gabbroic varieties. Thus, V/Sc and Fe^3+^/ΣFe for the two Proterozoic suites must be considered minima.

### Variable redox state of the Archaean convecting mantle

Foley^15^ suggested more reducing conditions for the Archaean convecting mantle and proposed that mantle eclogites may give useful constraints. This anticipation was confirmed by applying the V/Sc redox proxy to spreading ridge-derived (meta)basalts, which showed a significant difference between post-Archaean (∆FMQ-0.26 ± 0.44) and Archaean eclogite suites (∆FMQ-1.19 ± 0.33 2σ)^8^. The latter estimate is now considered a maximum, given recent experimental evidence that V behaves more incompatibly with increasing temperature^24^. Combined with secular mantle cooling, this implies that melts generated in the warmer Archaean mantle have higher V/Sc for a given redox state than those generated later in Earth’s history. Taking the temperature effect into account, many mantle and orogenic eclogites record ∆FMQ closer to −2 (Supplementary Fig. 2). The overall robustness of V during the evolution of mantle eclogites is strongly supported by recent work on deep-seated komatiite magmas, which were emplaced near continental margins and which never experienced seawater alteration, partial melting or mantle metasomatism. These samples show an oxidation trend across the Archaean-Palaeoproterozoic boundary of a similar magnitude (1.3 units in ∆logƒO_2_ (FMQ))^9,10^ as that obtained from eclogites (at least 1.0 unit)^8^, despite being based on an entirely different approach, namely partitioning of V between olivine or chromite crystals and komatiite melt vs. forward-modelling of V/Sc in a melt as a function of melt fraction and ƒO_2_.

Rather than consider V/Sc or Fe^3+^/ΣFe in isolation, as in previous studies, we here use the combined systematics in order to obtain new insights into the redox state of the Archaean mantle. Because accumulation and advanced differentiation lead to lower Fe^3+^/ΣFe and higher or lower V/Sc depending on ƒO_2_, respectively, relative to the little differentiated melt (see previous section), samples with evidence for either are excluded from consideration here, as are metasomatised samples. Both the reconstructed bulk-rock V/Sc and Fe^3+^/ΣFe for the remaining samples in two of the three Archaean suites (Orapa and Lace) are markedly and consistently low, with an average V/Sc of 5.07 ± 0.46 and Fe^3+^/ΣFe of 0.050 ± 0.015 (2σ), compared to MORB estimates of 6.8 ± 0.8 and 0.10 ± 0.02, respectively^8,25^. This attests to the significantly more reduced character of at least portions of the Archaean ambient convecting mantle. Despite having exclusively cumulate protoliths with minimum Fe^3+^/ΣFe and V/Sc as discussed in the previous section, unmetasomatised eclogites from both Diavik and Voyageur have higher V/Sc and Fe^3+^/ΣFe than Archaean ones with dominantly non-cumulate protoliths (Supplementary Dataset), and the average for the two Proterozoic suites is significantly higher (outside the 2σ uncertainty) than the average of the two reduced Archaean suites (Fig. 3b,c). Considering further that the convecting mantle has cooled through time leading to increasingly less incompatible behaviour of V^24^, the redox contrast in the source mantle that could be inferred from V/Sc systematics is even larger. This further underscores the postulated increase in convecting mantle *f*_O2_ across the Archaean-Proterozoic boundary^8–10^.

Interestingly, ca. 2.7 Ga eclogites from Koidu have higher average calculated bulk-rock V/Sc and Fe^3+^/ΣFe, both similar to modern MORB (Fig. 3c), which may indicate that part of the Archaean ambient mantle was oxidised to present-day levels. It may be significant that Koidu eclogites have on average higher FeO contents than the other Archaean eclogite suites (Fig. 2b), which cannot be explained by higher pressure of melting or by advanced degrees of differentiation during protolith formation^26^. Their source may be similar to that of coeval alkaline Fe-picrites from the Slave craton, which are characterised by elevated V/Sc (average 9.2) and were suggested to sample Fe-rich heterogeneities in the Archaean mantle that were melted out over time^27^. Redox heterogeneity is also recognised in komatiites where one Archaean suite yields modern MORB-like *f*_O2_^10^. Thus, Archaean eclogites and komatiites document chemical and redox heterogeneities, and locally oxidising conditions in the Archaean ambient convecting mantle, possibly reflecting differential upward mixing of post-core formation lower mantle that had been relatively oxidised by sequestration of Fe metal in the core^4,8^.

The consequences of a – predominantly – more reducing Archaean uppermost mantle are manifold. For example, it implies a decrease in the depth of redox melting (formation of CO_2_-bearing melt by oxidation of diamond in the asthenosphere^3^), which would have impeded stabilisation of carbonated melts beneath early-formed thick cratonic lithospheres. It further implies the release of a reducing volatile mix to the atmosphere, which helped impede the accumulation of atmospheric O_2_ prior to the Great Oxidation Event^8^.

### Low ƒO_2_ of deeply subducted Precambrian oceanic crust

**Figure 4:**
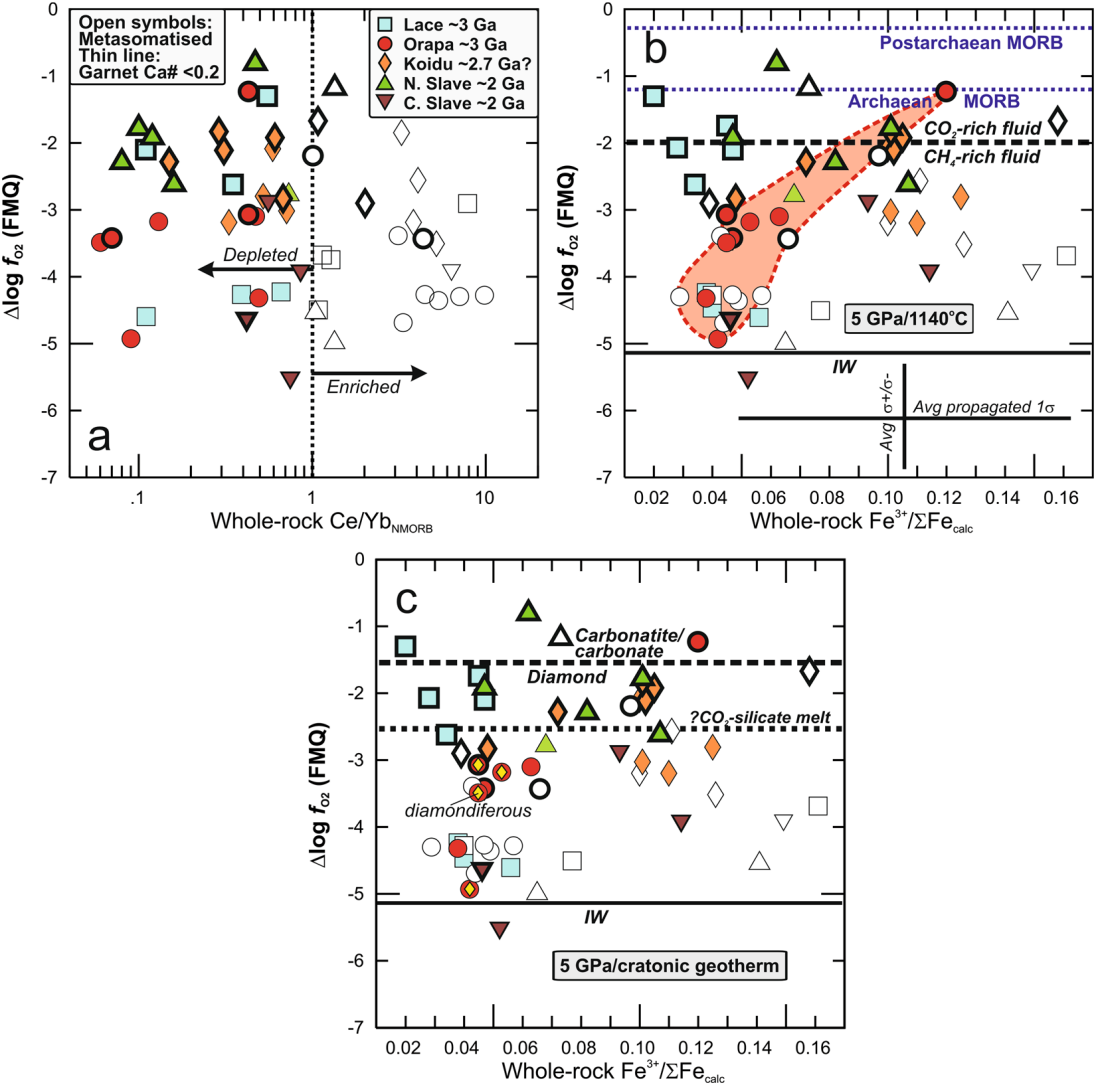
Oxygen fugacity estimates for mantle eclogite. Oxygen fugacity using the oxybarometer of^16^ and denoted as ∆logƒO_2_ (FMQ), i.e. calculated relative to the fayalite-magnetite-quartz buffer, as a function of (**a**) NMORB-normalised Ce/Yb in reconstructed whole rocks (as in Fig. 1) and (**b,c**) Fe^3+^/ΣFe in reconstructed whole rocks. Error bars on Fe^3+^/ΣFe reflect average propagated uncertainties as described in Fig. 1, on ∆logƒO_2_ they reflect average uncertainties propagated from those on garnet Fe^3+^/ΣFe (± 0.01). The symbols of samples with compositions far from the end-members on which the oxybarometer was formulated are shown with thin outline (see Methods). Thick stippled line in (**b**) separates CH_4_-dominated from CO_2_-dominated diamond-saturated fluid; ƒO_2_ corresponding to the iron-wuestite (IW) oxygen buffer is also shown (at 5 GPa and 1140 °C^31^). Thin blue stippled lines show ΔlogƒO_2_ of post-Archaean and Archaean MORB-equivalents with FMQ-0.26 ± 0.44 and FMQ-1.19 ± 0.33, respectively^8^. The majority of samples have lower ƒO_2_ than Archaean or modern MORB, requiring reduction of their protoliths upon subduction. Thick stippled line in (**c**) separates carbonatite melt or carbonate from graphite or diamond (at 5 GPa along a cratonic geotherm^13^). Most Palaeoproterozoic and Archaean eclogites at mantle depth would not be in equilibrium with pure carbonatite melt, but could exist with a carbonated silicate melt, by analogy with the peridotite system^3^. Diamondiferous Orapa eclogites are indicated by yellow diamonds.

Contrary to peridotite (e.g. ref. ^28^), there is no discernible effect of metasomatism on calculated ƒO_2_ in eclogite (Fig. 4a). Metamorphic reactions in addition to redox reactions involving the metasomatic oxidation or reduction of Fe may in part be responsible for the lack of correlation between ƒO_2_ and Fe^3+^/ΣFe in all suites except Orapa (Fig. 4b). A wide range of *f*_O2_ at a given pressure, even at a single locality, is also observed in peridotite xenoliths (Supplementary Fig. 1). In peridotites, this variability is explained by strong reduction upon initial melt extraction and subsequent interaction with metasomatic agents which can be both reducing (e.g. methane-dominated fluids) and oxidising (e.g. carbonated melts)^28^. In eclogites, it is ascribed to a superposition of intrinsic *f*_O2_ of metamorphosed oceanic crust, auto-metasomatic redox reactions occurring upon subduction, and metasomatism that also affects peridotites. These processes can also cause shifts in Fe^3+^/ΣFe, without completely resetting inherited crustal signatures, as discussed above. Even disregarding low-Ca samples with compositions far from those on which the oxybarometer was formulated (garnet Ca# < 0.2; Methods), it is evident that (1) eclogites have lower ƒO_2_ than either Archaean or modern MORB-like protoliths; (2) fluids in equilibrium with eclogite would be methane-dominated (Fig. 4b), with implications for the peridotite solidus temperature, which is higher in the presence of CH_4_ than of CO_2_^(1)^; (3) ƒO_2_ in the majority of eclogites (20 of 25) is too low to stabilise carbonate or allow percolation of pure carbonatite melt, which requires a ∆logƒO_2_ > ~FMQ-1.6 at 5 GPa (Fig. 4c). Rather, graphite or diamond is the stable carbon species.

Combined with Re-Os isotopic evidence for eclogitic diamond formation during Mesoarchaean craton amalgamation and Palaeoproterozoic lateral growth^29,30^ during collisional processes, these low eclogite ƒO_2_ suggest that oceanic crust represented an efficient trap for oxidised carbon in fluids and melts formed in ancient subduction environments. This not only helps explain the disproportionate frequency of eclogitic diamonds, relative to the subordinate abundance of eclogite in the mantle lithosphere^31^, but also provides support for carbon recycling at least to depths of diamond stability. Considering that the proportion of oceanic crust recycled to the sublithosphere through time far exceeds that which was captured in the continental lithosphere, diamond formation in reducing subducting oceanic crust may have represented an efficient pathway for carbon ingassing upon deep subduction, consistent with the observation that 35–80% of C has been recycled from the exosphere to the deep mantle^32^. In contrast to pure carbonatite, the *f*_O2_ of a higher proportion of eclogites investigated here would be permissive of percolation of a carbonated silicate melt, such as kimberlite. A kimberlite-like melt containing 10% CO_2_ would be stable to lower ƒO_2_ by ~1 log unit, compared to pure carbonatite melt, by analogy with the peridotite system^3^. This redox “window” allows for the precipitation of additional diamond in mantle eclogite, by reduction of CO_2_ in kimberlite-like melts.

Mantle eclogites metasomatised after their incorporation into the cratonic lithosphere, either cryptically (identified by high Ce/Yb) or modally (such as phlogopite-bearing eclogites^23^), are not representative of oceanic crust recycled into the convecting mantle. We suggest that the *f*_O2_ of the remaining samples is so low that distributed carbonate grains or carbonate pockets in seawater-altered oceanic crust (as opposed to carbonate sediments) are likely to be reduced to diamond upon subduction. Thus, the inferred (from experiments) or demonstrated (from inclusions in diamonds) presence of CO_2_-dominated fluids or carbonatite in the convecting mantle (e.g. ref. ^33^) cannot be explained by appealing to subduction of seawater-altered oceanic crust except possibly subsequent to Neoproterozoic oxygenation of oceanic bottom waters^20^. For older convecting mantle sources, recycling of carbonate-rich sediments may be required, the oxidising power of which can regionally overwhelm the buffering capacity of the dominantly reducing convecting mantle and the eclogitic/pyroxenitic heterogeneities it contains.

## Methods

### Oxygen isotope analysis by secondary ion mass spectrometry (SIMS)

Sample preparation and analysis by SIMS were carried out at the Canadian Centre for Isotopic Microanalysis (CCIM), University of Alberta. Garnet mineral separates were mounted with CCIM garnet reference materials (RMs) S0068 (Gore Mountain Ca-Mg-Fe garnet) and S0088B (grossularite) and exposed in a 25 mm diameter epoxy assembly (M1506) using diamond grits. The mount was cleaned with a lab soap solution and de-ionized H_2_O, and then coated with 20 nm of high-purity Au prior to scanning electron microscopy (SEM). SEM characterization was carried out with a Zeiss EVO MA15 instrument using beam conditions of 20 kV and 3–4 nA. A further 80 nm of Au was subsequently deposited on the mount prior to SIMS analysis.

Oxygen isotope ratios (^18^O/^16^O) in garnet from Orapa, Koidu and Diavik were determined with a Cameca IMS 1280 multicollector ion microprobe, using previously described analytical methods and reference materials^34^. Briefly, a ^133^Cs^+^ primary beam was operated with an impact energy of 20 keV and beam current of ~2.0–2.5 nA. The ~12 µm diameter probe was rastered (20 × 20 µm) for 30 s prior to acquisition, and then 8 × 8 µm during acquisition. Negative secondary ions were extracted through 10 kV potential into the secondary (Transfer) column. All regions of the sputtered area were transferred and no energy filtering was employed. The mass/charge-separated oxygen ions were detected simultaneously in Faraday cups with 10^10^ Ω (^16^O^−^) and 10^11^ Ω (^18^O^−^) amplifier circuits, respectively. A single analysis took 240 s, including pre-analysis primary beam implantation, automated secondary ion tuning, and 75 s of continuous peak counting. Instrumental mass fractionation (IMF) was monitored by repeated analysis of S0068 (UAG) and S0088B (δ^18^O_VSMOW_ =  + 5.72‰ and + 4.13‰, respectively), with one analysis of S0068 and S0088B taken after every 4 and 8 unknowns, respectively. The data set of ^18^O^−^/^16^O^−^for S0068 garnet yielded standard deviations of 0.09‰ and 0.08‰, respectively, for each of two analytical sessions and after correction for systematic within-session drift (≤ 0.4‰). Data for S0088B and unknowns were first IMF-corrected to S0068 garnet, and then further corrected according to their measured Ca# (Ca/[Ca + Mg + Fe]) using the methods outlined by Ickert and Stern^34^. The average 95% confidence uncertainty estimate for δ^18^O_VSMOW_ for garnet unknowns is ± 0.30‰ and includes errors relating to within-spot counting statistics, geometric effects, correction for IMF, and matrix effects relating to Ca# determined by electron microprobe.

### Fe^3+^/ΣFe in garnet ± cpx by Mössbauer spectroscopy

The sample preparation and analytical routine for the determination of Fe^3+^/ΣFe in garnet and cpx by Mössbauer spectroscopy at Goethe-University Frankfurt, employing a nominally ~50 mCi ^57^Co in Rh source, has been described in^12^. Briefly, handpicked, optically clean mineral separates were powdered under acetone and packed into a hole drilled in 1 mm thick Pb discs. To minimise saturation effects, the amount of sample and hole diameter were chosen such that a sample thickness of < 5 mg Fe cm^−2^ was obtained. To this end, when necessary, a small amount of sugar was mixed with the mineral powder to create a uniform sample that filled the volume of the drilled hole. This also serves to limit any preferred orientation in the sample that might influence the spectrum. ^57^Fe spectra were collected until a target value of > 2 × 10^6^ background counts was achieved (representative Mössbauer spectra in Supplementary Fig. 3). Recoil-free fraction effects were corrected as given by^35^. Uncertainties on Fe^3+^/ΣFe are typically ± 0.01 absolute.

### Fe-based oxybarometry

Oxygen fugacity, reported as ∆logƒO_2_ relative to FMQ (Fayalite-Magnetite-Quartz buffer; e.g.^36^), was calculated with a new thermodynamic formulation of the oxybarometer for eclogites by^16^. Details on this barometer are provided in^12^, with additional information in an unpublished PhD thesis that is available in an online repository (link provided in reference list). Although this thesis has undergone and passed examination, we recognise that the lack of peer review may raise doubts regarding use of the new oxybarometer. We thus emphasise that the underlying principles are identical to those in a published barometer^13^, and that the conclusions reached in this study are independent of which oxybarometer is used. The two oxybarometers return highly correlated values, but results according to^13^ are off-set towards lower values (Supplementary Fig. 5), which would require metal saturation in some samples to occur and is inconsistent with petrographic observations. We therefore prefer to report values according to^16^). We use the iteratively calculated pressures and temperatures (Supplementary Text), mineral compositions as well as garnet Fe^3+^/ΣFe displayed in Supplementary Dataset 1 as input parameters. The oxybarometer is based on activities of garnet solid solution end-members and the hedenbergite component in cpx in equilibrium with a SiO_2_ phase, as follows:

1 $$\begin{array}{lllllllll}5\,{{\rm{CaFe}}}^{2+}{{\rm{Si}}}_{2}{{\rm{O}}}_{6} & + & 1/3\,{{\rm{Ca}}}_{3}{{\rm{Al}}}_{2}{{\rm{Si}}}_{3}{{\rm{O}}}_{12} & +\,{{\rm{O}}}_{2} & = & 2{{\rm{Ca}}}_{3}{{\rm{Fe}}}_{2}^{3+}{{\rm{Si}}}_{3}{{\rm{O}}}_{12} & + & 1/3\,{{\rm{Fe}}}_{3}^{2+}{{\rm{Al}}}_{2}{{\rm{Si}}}_{3}{{\rm{O}}}_{12} & +\,4\,{{\rm{SiO}}}_{2}\\ {\rm{hedenbergite}}\,{\rm{in}}\,{\rm{cpx}} &  & {\rm{grossular}}\,{\rm{in}}\,{\rm{garnet}} &  &  & {\rm{andradite}}\,{\rm{in}}\,{\rm{garnet}} &  & {\rm{almandine}}\,{\rm{in}}\,{\rm{garnet}} & {\rm{coesite}}\end{array}$$

Although coesite is absent in all but one sample from Koidu, the effect is expected to be minor (for example, a_SiO2_ = 0.85 instead of 1.0 translates into a shift in ∆logƒO_2_ of ~−0.3 log units), except under strongly SiO_2_-undersaturated conditions when corundum would be present^12^, a mineral that is not observed in the sample suite under investigation. Also, at low a_SiO2_ a significant Tschermaks component would be expected in cpx, which is in conflict with the observed occupancy of essentially 2 Si cations per formula unit (c.p.f.u.). In addition to uncertainty related to the thermobarometer formulation itself (~60 °C), lack of equilibrium to the regional geotherm, for example due to melt-advected heat, entails that pressures would also be overestimated; a temperature uncertainty of 100 °C translates into a pressure difference of 0.7 GPa along a conductive geotherm and an uncertainty in ∆logƒO_2_ of 0.23 log units^12^. The largest source of error remains the precision with which Fe^3+^/ΣFe can be determined, which corresponds to ± 0.01, and for very low absolute Fe^3+^/ΣFe can be very large and asymmetric^16^, corresponding to average 1σ + 0.84/ −0.81. Additional uncertainty is introduced when the oyxbarometer is applied to samples with compositions far from those on which it was formulated (see equation above). There is no geological explanation (differentiation, metasomatism etc.) for the vague and marked positive trend of ƒO_2_ with jadeite mole fraction and Ca# of garnet, respectively, in Supplementary Fig. 4, which may indicate that samples with grossular-poor garnet, which are furthest from the end-member compositions on which the oxybarometer was formulated, yield underestimated ƒO_2_. However, it is also clear that one of the central Slave samples with high garnet Ca# nevertheless yields very low ∆logƒO_2_ and that there is a marked positive correlation between Fe^3+^/ΣFe and ∆logƒO_2_ in Orapa samples (Fig. 4b), including those with low Ca#, suggesting that the relationship between Ca# and ∆logƒO_2_ is not straightforward to interpret. In the interest of caution, we indicate samples with garnet Ca# < 0.2 and only discuss ƒO_2_ for samples with higher values.

### Bulk-rock reconstruction of Fe^3+^/ΣFe and V/Sc

The distribution *D* of Fe^3+^/ΣFe between cpx and garnet varies between 3.6 and 20. Increasing temperature leads to increased partitioning of Fe^3+^ into garnet at the expense of cpx^12^ and this is also the case for garnet-cpx pairs in Orapa eclogites (Supplementary Fig. 6). In addition, garnet Fe^3+^/ΣFe is higher in high-temperature than in low-temperature eclogites from Lace, Koidu and the central Slave craton, with no temperature-dependence observed for northern Slave eclogites (not shown). Large scatter is evident at low temperatures, where the xenolith population is dominated by metasomatised (LREE-enriched) samples. Clinopyroxene in metasomatised samples tends to be jadeite-poor, and Fe^3+^ partitioning into jadeite-poor cpx is reduced, as evident from Supplementary Fig. 6, while partitioning into garnet is enhanced. Thus, there is a superposition of temperature and crystal-chemical effects. To mitigate the latter, we focus on samples with jadeite mole fractions ≥ 0.27. The resultant regression (Supplementary Fig. 6) has a large uncertainty on the slope and the intercept. Removal of two visual outliers does not significantly change the slope or intercept of the regression. Propagating the ± 0.01 uncertainty on the Mössbauer-derived Fe^3+^/ΣFe in cpx and in garnet, the average resultant uncertainty on ^cpx/garnet^D(Fe^3+^/ΣFe) is ± 5.6. The regression allows calculation of Fe^3+^/ΣFe in cpx as a function of temperature (Supplementary Dataset) and garnet Fe^3+^/ΣFe, which yields Fe^3+^/ΣFe from 0.06 to 0.33 in samples from this and published studies. Propagating the uncertainty on the slope (±0.0039) and on the intercept (±6) of the regression results in an average uncertainty on the calculated Fe^3+^/ΣFe in cpx of ± 0.28. Measured and calculated Fe^3+^/ΣFe in Orapa cpx are compared in Supplementary Fig. 6.

Whole rock reconstruction is standard procedure for eclogite xenoliths to avoid kimberlite contamination. The coarse grain size combined with typically small sample size precludes accurate modal determination. Modal abundances of 55% garnet and 45% cpx are considered appropriate for eclogites with picritic protoliths^14^. These values are corroborated by average modal abundances measured in exceptionally large xenoliths (with 1σ of ~5%) and modes determined for experimental subsolidus assemblages in mafic systems where variations as a function of pressure are ~8% (see discussion in^26^). Here, a blanket uncertainty of 10% is assumed. For Orapa, bulk rock Fe^3+^/ΣFe was reconstructed by weighting measured garnet and cpx Fe^3+^/ΣFe by the wt% Fe contributed by each mineral and applying the aforementioned modal abundances. Propagation of a 5% uncertainty on the garnet and cpx mode each (for a total estimated uncertainty of 10%), as well as the 0.01 uncertainty on mineral Fe^3+^/ΣFe as obtained by Mössbauer spectrometry, results in an average uncertainty on the Orapa bulk-rock Fe^3+^/ΣFe of 0.011. For the remaining samples, bulk rocks were reconstructed using the same modes plus uncertainties from measured garnet Fe^3+^/ΣFe and calculated cpx Fe^3+^/ΣFe. Propagation of (1) the uncertainties on their respective Fe^3+^/ΣFe, (2) a 5% uncertainty each on the garnet and cpx mode, weighted by (3) the contribution of each mineral to the calculated whole rock Fe content by weight results in average uncertainties of ± 0.057. Despite the large uncertainties on the Fe^3+^/ΣFe of the calculated cpx, its contribution to the whole rock Fe_total_ content is minor (average ~20%). This explains the comparatively low uncertainty on the calculated whole rock, which is dominated by garnet and the much lower uncertainty on its measured Fe^3+^/ΣFe, though sizable in terms of absolute value relative to the very low Fe^3+^/ΣFe determined. Results for Orapa whole rocks using measured and calculated cpx Fe^3+^/ΣFe are compared in Supplementary Fig. 6.

Eclogitic bulk elemental compositions are also reconstructed from mineral compositions weighted by modes assuming 55% garnet and 45% cpx. Mantle eclogite minerals often have very homogeneous compositions (low standard deviations for multiple analyses per sample), and V and Sc are present at concentrations far above the detection limit; since V partitions more strongly into cpx than into garnet, increasing the cpx mode by 10% will lead to an increase in V/Sc of the calculated bulk rock by < 1 ^8^. Furthermore, rutile, which is a frequent accessory mineral in mantle eclogite, but not always exposed in sections, contains 100 s to 1000 s ppm of V (Aulbach, unpubl. database). Rutile modes are estimated by assuming that Ti is not depleted relative to Sm and Gd, as applies to melts from subduction-unmodified sources^14^, and reported in the Supplementary Dataset. Here, bulk rock V was calculated by considering the measured V concentrations in garnet, cpx and assuming a median V concentration in mantle eclogite rutile of 1270 ppm (Aulbach, unpubl. database). This leads to a small average increase in V/Sc from 5.99 to 6.06. Assuming median FeO contents measured in rutile from Koidu are representative (0.96 wt%, n = 28)^26^, the proportion of FeO controlled by rutile is minute (<0.05; Supplementary Dataset) and is not further considered.

To estimate average “primary” Fe^3+^/ΣFe and V/Sc for various sample suites displayed in Fig. 3c, only eclogites and pyroxenites with non-cumulate protoliths that did not experience high degrees of differentiation are considered, which excludes gabbroic and high-Ca eclogites^14^. In addition, metasomatised samples with Ce/Yb_NMORB_ > 1 are excluded, while melt-depletion from eclogite has no discernible effects on the two redox proxies employed, as discussed in the main text. This yields average estimates of Fe^3+^/ΣFe and V/Sc for 13 and 4 samples, respectively, from Koidu, 4 and 3 samples, respectively, from Orapa, and 22 and 3 samples, respectively, from Lace. Eclogites from Voyageur and Diavik in the northern and central Slave craton, respectively, have a strong cumulate character and are therefore considered unrepresentative of melts.

## Supplementary information

Supplementary Information

Supplementary Information 2
