# Supplementary material for: Evidence for a dominantly reducing Archaean ambient mantle from two redox proxies, and low oxygen fugacity of deeply subducted oceanic crust
Source: Sci Rep. 2019 Dec 27;9:20190. doi: 10.1038/s41598-019-55743-1 (PMC6934757; doi:10.1038/s41598-019-55743-1)
Supplement: Supplementary file 1 — Supplementary Information [file 41598_2019_55743_MOESM1_ESM.pdf]

Supplementary information for

Evidence for a dominantly reducing Archaean ambient mantle from two redox proxies, and low oxygen fugacity of deeply subducted oceanic crust

Sonja Aulbach, Alan B. Woodland, Richard A. Stern, Prokopiy Vasilyev, Larry M. Heaman, K. S. Viljoen

This file includes:

Supplementary Text 1. Background information on samples and crystal-chemical controls on Fe^{3+} incorporation into eclogite minerals.

Supplementary Figure 1. Estimated oxygen fugacities as a function of depth.

Supplementary Figure 2. Estimated oxygen fugacities of Archaean eclogites based on V-Ti relationships

Supplementary Figure 3. Mössbauer spectra.

Supplementary Figure 4. Estimated oxygen fugacities in mantle eclogite vs. mineral compositions.

Supplementary Figure 5 Estimated oxygen fugacities according to two eclogite oxybarometers.

Supplementary Figure 6. Distribution of $Fe^{3+}/\Sigma Fe$ between garnet and clinopyroxene (cpx).

Supplementary Figure 7. REE relationships in mantle eclogites and MORB.

Supplementary Figure 8. $Fe^{3+}/\Sigma Fe$ and elemental relationships in mantle eclogites.

Supplementary Figure 9. Effects of crystal chemistry and metasomatism on $Fe^{3+}/\Sigma Fe$ in cpx.

Supplementary Figure 10. Crystal-chemical controls on $Fe^{3+}/\Sigma Fe$ in garnet.

Supplementary Text 1

Samples – further details. The study comprises kimberlite-borne mantle eclogite and pyroxenite xenoliths from Orapa (Zimbabwe craton), Koidu (West African craton) and Diavik (central Slave craton). Details on their petrogenesis and on the geological background relevant to the origin and evolution of the cratonic lithospheric mantle in which they are hosted are provided in (*39-40*) for Orapa, (*26*) for Koidu and (*41*) for Diavik. Samples are classified according to ref. (14). Samples cannot be classified in this scheme if major or trace element data are unavailable for garnet and/or clinopyroxene, as applies to a few samples in this study for which garnet $Fe^{3+}/\Sigma Fe$ was determined.

Seventeen samples representing the main compositional types from Orapa were chosen for this study. Prior trace-element investigation (Aulbach, unpubl. data) reveals that six are gabbroic eclogites (Eu/Eu* >1.05, therefore having plagioclase-rich cumulate protoliths), two high-Ca eclogites and four low-Mg eclogites with variably differentiated igneous protoliths, in addition to four pyroxenites with jadeite-poor cpx. One sample (801) consists entirely of cpx with a jadeite content of 0.34 and Eu/Eu* of 0.97 and is therefore eclogitic rather than gabbroic or pyroxenitic. This sample and samples 787, 789, 790 and 794 are diamondiferous whereas the remainder is barren. Temperature and pressure estimates were obtained by iterative solution of the regional peridotite-derived geotherm (using a 41 mW/m^2 surface heat flow which returns pressures for all samples within the lithosphere) with the temperatures calculated using the thermometer of (*42*), which yields 640 to 1610°C and 2.0 to 7.2 GPa (Supplementary Dataset 1). High-Mg eclogites and pyroxenites often have elevated NMORB-normalised Ce/Yb (as a proxy of metasomatism), low jadeite contents in cpx and low equilibration temperatures (Supplementary Dataset 1). The Ce/Yb ratio is broadly negatively correlated with Yb/Gd in mantle eclogite because Yb is more compatible than Gd during

partial melt extraction from eclogite[14], and ranges to much lower Ce/Yb and higher Yb/Gd than NMORB, hence justifying the normalisation (Supplementary Fig. 7).

Garnet separates from 16 representative samples from Koidu were prepared for the present study. They encompass high-Ca (n=6), low-Mg (n=1) and high-Mg (n=6) eclogites, one gabbroic eclogite and one gabbroic pyroxenite. Similar to Orapa, four high-Mg eclogites with MgO >17 wt% are metasomatised (Ce/Yb_N >1), as are an additional two samples with lower MgO content. Temperature and pressure estimates were obtained as described above using the cpx xenocryst-derived geothermal gradient corresponding to 38 mW/m^2 surface heat flow (*43*). They range from 840 to 1320°C and from 3.6 to 6.6 GPa, respectively. One sample (K5-13) apparently last equilibrated at 1510°C (7.9 GPa), corresponding to sub-lithospheric depth, which either reflects disequilibrium or late heating and failure to equilibrate to the geotherm (Supplementary Dataset 1).

Garnet separates were prepared for five samples from Diavik previously investigated by (*41*), with a more complete list of trace element acquired in-house (Aulbach, unpubl. data). They all have positive Eu anomalies and encompass 3 gabbroic eclogites plus 2 gabbroic pyroxenites, one of the latter being metasomatised (Ce/Yb_{NMORB} >1). They give iteratively calculated temperatures from 810 to 1420°C and pressures from 3.5 to 7.3 GPa (Supplementary Dataset 1).

Crystal-chemical controls on Fe^{3+} incorporation in garnet and cpx. As a de facto trace element with a compatibility between that of Li and In[6], the incorporation of Fe^{3+} in eclogitic garnet and cpx is expected to reflect crystal-chemical control, which is also related to temperature and bulk composition. Indeed, there is a vague correspondence between In and

$Fe^{3+}/\Sigma Fe$ for unmetasomatised reconstructed whole rocks (Supplementary Fig. 8A). None is observed for Li, which can be explained by the susceptibility of this element to seawater alteration and its loss during metasomatism[26], which also appears to affect In (Supplementary Fig. 8A-B). For the Orapa suite, where both garnet and cpx were measured, the various controls on Fe^{3+} uptake are difficult to disentangle because metasomatised samples not only have lower jadeite contents but also have equilibrated at lower temperatures than non-metasomatised samples. There is no relationship of Fe^{3+} per formula unit (pfu) with temperature, but higher values are observed for cpx with high Na_2O content, expressed as jadeite mole fraction, which may indicate coupled substitution of Fe^{3+} with Na for two divalent cations in the mineral structure (Supplementary Fig. 9A-B). Metasomatised samples not only have lower jadeite content but also lower Fe^{3+} pfu than non-metasomatised ones (Supplementary Fig. 9C-D). A plot of Fe^{3+} pfu as a function of total Fe pfu reveals that, for a given total Fe content, cpx in metasomatised samples has higher Fe^{3+} than that in unmetasomatised ones (Supplementary Fig. 9E).

For garnet in all three eclogite suites, $Fe^{3+}/\Sigma Fe$ increases with increasing temperature (Supplementary Fig. 10A). This is also observed for garnet in peridotite xenoliths (e.g.[44]), but the relationship is lost in some suites and weaker for Orapa and Lace eclogites if the Fe^{3+} content pfu is considered (Supplementary Fig. 10B). Negative correlations between Fe^{3+} pfu and the trivalent cations Al and Cr would indicate competitive element substitution behaviour, while a positive correlation with Na would suggest sympathetic behaviour due to coupled substitution, as previously described for Lace[12] and for peridotitic assemblages[44-45]. However, such relationships are weak at best in the samples under consideration (Supplementary Fig. 10C-E). As shown for cpx, metasomatism tends to induce low total Fe contents. Higher Fe^{3+} in garnet from metasomatised samples for a given Fe_{total} is only observed for the Lace and

Voyageur suites (Supplementary Fig. 10F). An anticorrelation between Fe^{3+} in garnet and its Ca#, described before for eclogite xenoliths from the northern Slave craton[46] and Lace[12], is not evident for other eclogite suites in this study, while Orapa shows a positive correlation (Supplementary Fig. 10H).

Finally, it is known that increasing bulk-rock Ca content, expressed as Ca# in garnet, facilitates the incorporation of certain trace elements (Sr, Y, REE; e.g.[47]), but there is no clear correspondence between high garnet Ca# and increasing partitioning of Fe^{3+} (Supplementary Fig. 8C), although this is observed for In (Supplementary Fig. 8D). Thus, Fe^{3+} does not unambiguously behave like the similarly incompatible trace element In[6] with respect to its distribution between cpx and garnet. The sometimes contrasting behaviour of cpx and garnet from different eclogite suites, and the frequent lack of clear correlations between Fe^{3+}, temperature and composition, are ascribed to superposition of the effects these parameters have on the exchange of Fe^{3+} between garnet and cpx.

Additional references

39. Viljoen, K. S., Smith, C. B. & Sharp, Z. D. Stable and radiogenic isotope study of eclogite xenoliths from the Orapa kimberlite, Botswana. *Chem. Geol.* 131, 235-255 (1996).
40. Aulbach, S., Jacob, D. E., Cartigny, P., Stern, R. A., Simonetti, S. S., Wörner, G. & Viljoen, K. S. Eclogite xenoliths from Orapa: Ocean crust recycling, mantle metasomatism and carbon cycling at the western Zimbabwe craton margin. *Geochim. Cosmochim. Acta* 213, 574-592 (2017).
41. Schmidberger, S. S., Simonetti, A., Heaman, L. M., Creaser, R. A. & Whiteford, S. Lu-Hf, in-situ Sr and Pb isotope and trace element systematics for mantle eclogites from the Diavik diamond mine: Evidence for Paleoproterozoic subduction beneath the Slave craton, Canada. *Earth Planet. Sci. Lett.* 254, 55-68 (2007).
42. Krogh Ravna, E. J. The garnet-clinopyroxene Fe^{2+}-Mg geothermometer: an updated calibration. *J. Metam. Geol.* 18, 211-219 (2000).
43. Smit, K. V., Shirey, S. B. & Wang W. Y. Type Ib diamond formation and preservation in the West African lithospheric mantle: Re-Os age constraints from sulphide inclusions in Zimmi diamonds. *Precamb. Res.*, 286, 152-166 (2016).

44. Woodland, A. B. Ferric iron contents of clinopyroxene from cratonic mantle and partitioning behaviour with garnet. *Lithos* 112, 1143-1149 (2009).
45. Malaspina, N., Langenhorst, F., Fumagalli, P.m Tumiati, S. & Poli, S. Fe^{3+} distribution between garnet and pyroxenes in mantle wedge carbonate-bearing garnet peridotites (Sulu, China) and implications for their oxidation state. *Lithos* 146, 11-17 (2012).
46. Kopylova, M. G., Beausoleil, Y., Goncharov, A. Burgess, J. & Strand, P. Spatial distribution of eclogite in the Slave cratonic mantle: The role of subduction. *Tectonophys.* 672, 87-103 (2016).
47. Aulbach, S. Gerdes, A. & Viljoen, K. S. Formation of diamondiferous kyanite-eclogite in a subduction melange. *Geochim. Cosmochim. Acta* 179, 156-176 (2016).
48. Jenner, F. E. & O'Neill, H. S. Major and trace analysis of basaltic glasses by laser-ablation ICP-MS. *Geochem. Geophys. Geosyst.* 13, doi:10.1029/2011gc003890 (2012).
49. Aulbach, S. & Arndt, N. T. Eclogites as Palaeodynamic Archives: Evidence for Warm (not hot) and Depleted (but heterogeneous and evolving) Archaean Ambient Mantle. *Earth Planet. Sci. Lett.* 505, 162-172 (2019)
50. Sun, S.-s. & McDonough, W. F. Chemical and isotopc systematics of oceanic basalts: implications for mantle composition and processes. In A. D. Saunders, M. J. Norry (Eds.*), Magmatism in the Ocean Basins* (pp. 313-345). London: Geological Society.

Supplementary Figures 1-10

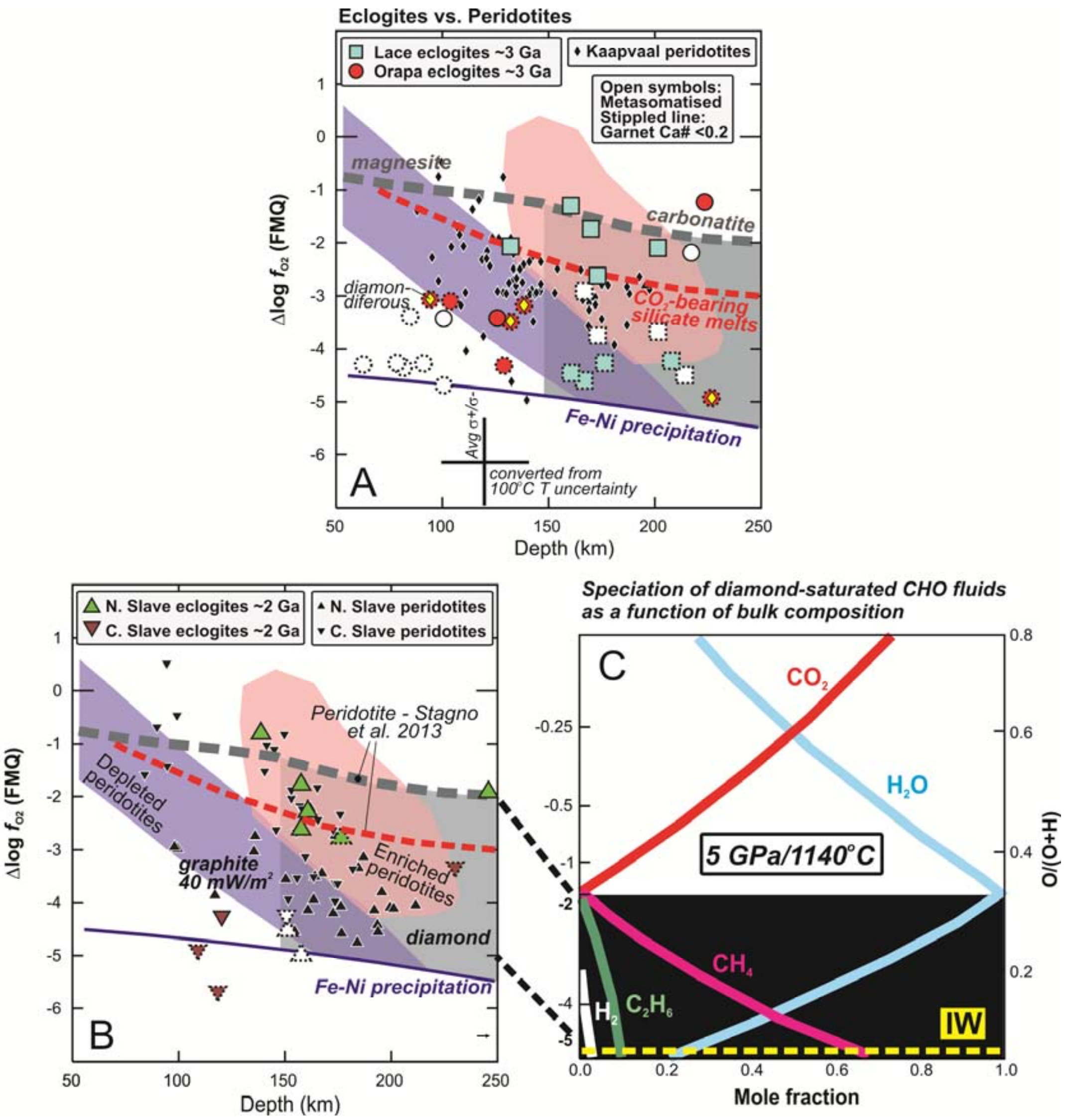

Supplementary Figure 1. Estimated oxygen fugacities as a function of depth. Oxygen fugacity relative to the Fayalite-Magnetite-Quartz (FMQ) buffer is expressed as $\Delta \log fO_2$ (FMQ); depth was converted from pressure derived by iterative solution of calculated temperatures with regional geotherms, as described in the Supplementary Text. Symbols of samples with low garnet Ca#, that is, compositions far from the end-members on which the oxybarometer was formulated (Methods), are shown with stippled outline. In (**A**) results for Lace and Orapa and in (**B**) for northern and central Slave eclogites are compared to peridotite-derived fO_2 from the Kaapvaal and Slave craton, respectively (Woodland, unpubl. database), to illustrate that the dominantly peridotitic subcontinental lithospheric mantle does not impose its fO_2 on volumetrically subordinate eclogite. Stability fields for carbonate (magnesite) vs. graphite or diamond (coexisting at ~150 km for a conductive geotherm corresponding to a surface heat flow of 40 mW/m^2), for carbonated silicate melt with 10% CO_2 (molar) and Fe-Ni precipitation curve from (*3*). Fields for enriched vs. depleted peridotites from (*28*). In (A) the error cross shows average 1 sigma uncertainty derived as detailed in Methods. Metasomatism is gauged by NMORB-normalised bulk-rock Ce/Yb >1 (NMORB of *37*). (**C**) Speciation of diamond-saturated CHO fluids at 5 GPa and 1140°C, showing $\Delta \log fO_2$ (FMQ) (note non-linear scale) and O/(O+H) as a function of mole fraction volatile species as well as the location of the iron-wuestite (IW) oxygen buffer (after *31*).

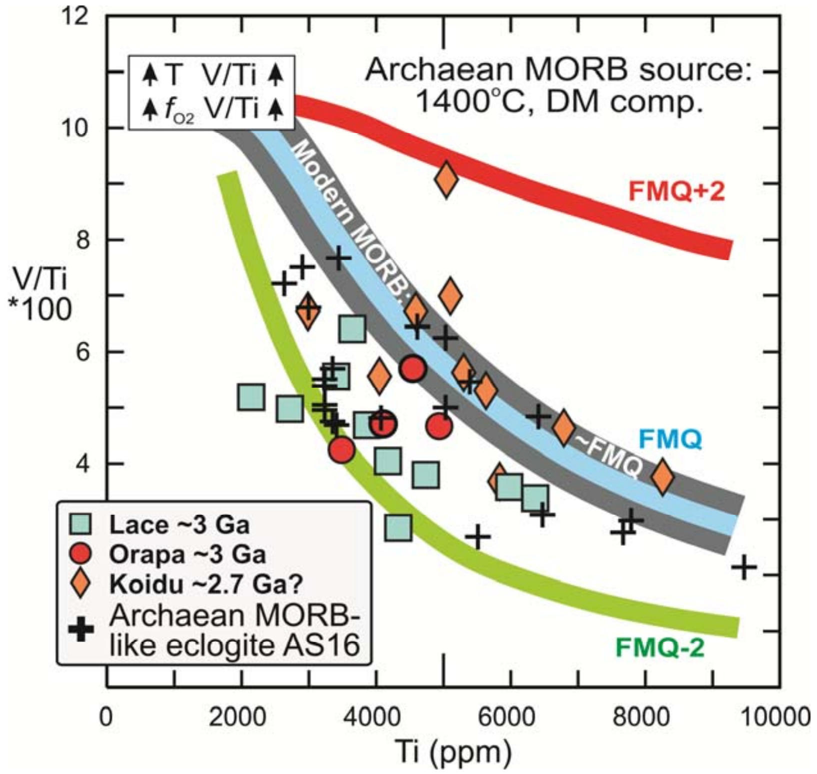

Supplementary Figure 2. Estimated oxygen fugacity in eclogites based on V-Ti systematics. Mantle eclogite xenoliths investigated in this study plus other Archaean mantle and orogenic eclogites ("AS16") described in (*8*) are plotted in Ti vs. Ti/V*100 space with fO_2 isopleths relative to the Fayalite-Magnetite-Quartz (FMQ) buffer derived by (*24*) as a function of temperature, here assuming very moderate Archaean mantle potential temperature of 1400 °C, and composition, here assuming a Depleted Mantle (DM) source[49]. Only samples with non-cumulate, non-metasomatised, little differentiated protoliths are shown that can be reasonably compared to forward models of peridotite-derived melts. At higher temperatures at a given fO_2, V behaves more incompatibly[24] such that the isopleths would be shifted upwards, implying even more reducing conditions in parts of the Archaean mantle compared to modern MORB. The latter was estimated by Wang et al. (*24*) to be around FMQ based on the same approach but for modern mantle potential temperatures of 1300 °C (thick grey curve).

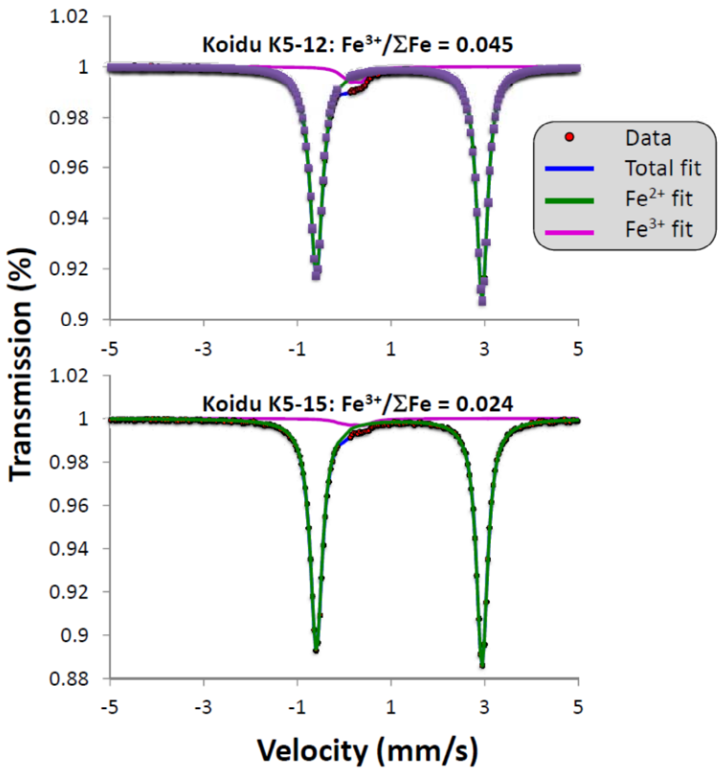

Supplementary Figure 3. Mössbauer spectra. Representative Mössbauer spectra of garnet in two eclogite xenoliths from Koidu, West African craton, with different $Fe^{3+}/\Sigma Fe$.

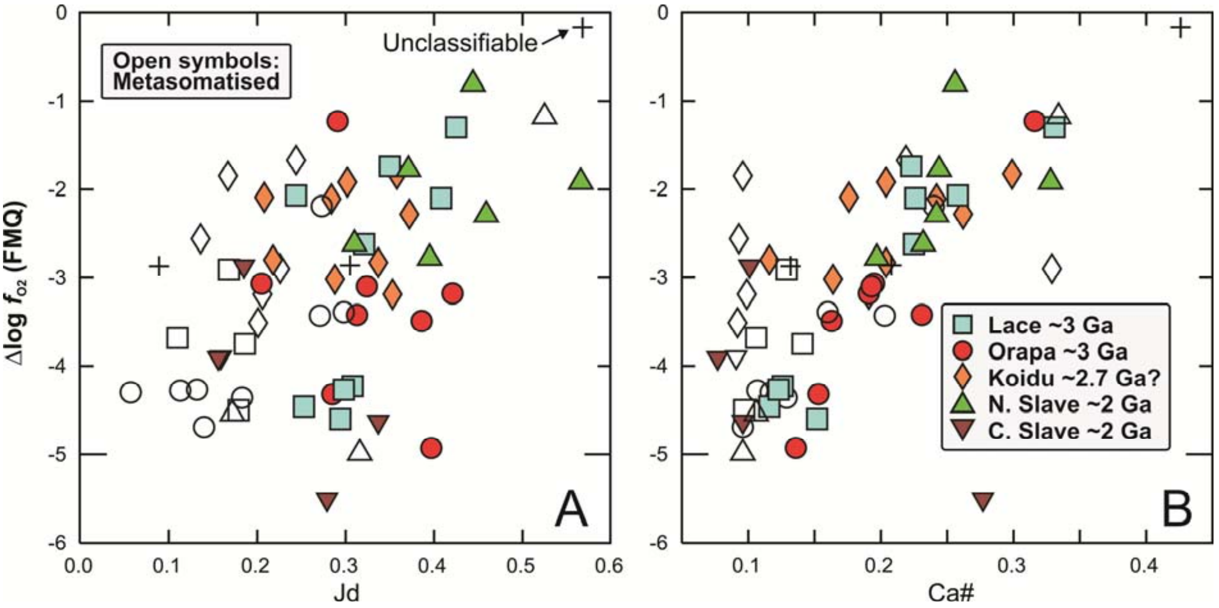

Supplementary Figure 4. Estimated oxygen fugacity in mantle eclogite vs. mineral compositions. Oxygen fugacity relative to the Fayalite-Magnetite-Quartz (FMQ) buffer, expressed as $\Delta \log f O_2$ (FMQ) as a function of (**A)** jadeite content in cpx (Jd) and (**B)** Ca# (Ca/(Ca+Fetotal+Mg+Mn) molar) in garnet. Open symbols distinguish samples that have been metasomatised, as gauged by NMORB-normalised bulk-rock Ce/Yb >1 (NMORB of *37*). Some samples are unclassifiable because relevant clinopyroxene and/or garnet data are unavailable.

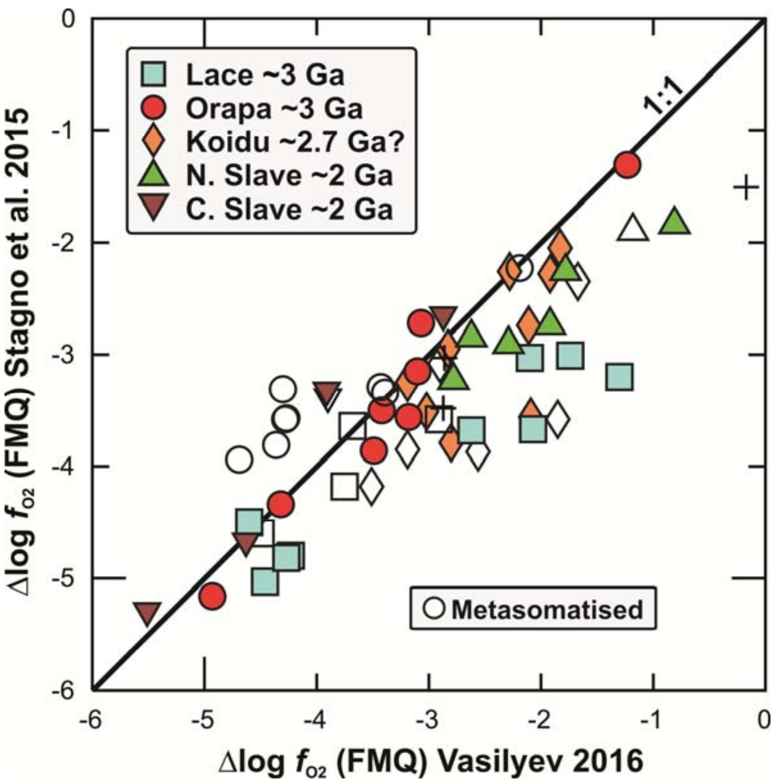

Supplementary Figure 5. Comparison of Δlog$f$O$_2$ results returned by the oxybarometer of Vasilyev (*16*) vs. that of Stagno et al. (*13*). Calculations were carried out as described in Methods. Values are highly correlated, though offset to lower Δlog$f$O$_2$ for the latter. For reasons outlined in Methods, we prefer to show Δlog$f$O$_2$ calculated according to (*15*). Metasomatism is gauged by NMORB-normalised bulk-rock Ce/Yb >1 (NMORB of *37*).

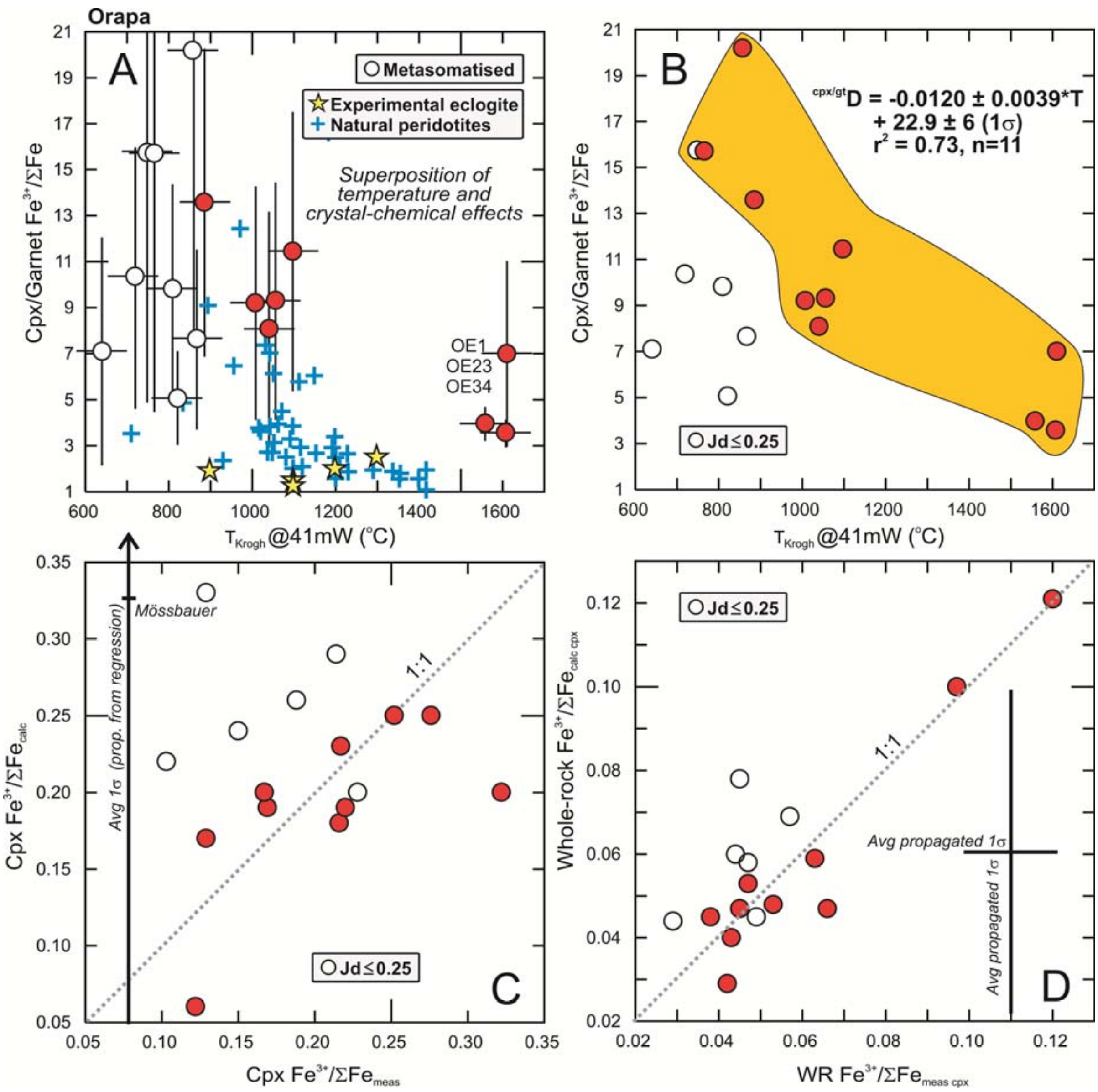

Supplementary Figure 6. Distribution of $Fe^{3+}/\Sigma Fe$ between garnet and cpx. (A) Ratio of $Fe^{3+}/\Sigma Fe$ in cpx over that in garnet as a function of temperature (derived as described in Supplementary Text). Error bars on x-axis reflect 60°C uncertainty on temperature estimates[42], neglecting potential errors due to lack of equilibration to the regional geotherm; on the y-axis they reflect the propagated uncertainty on Mössbauer determinations of ±0.01. Open symbols distinguish samples that have been metasomatised, as gauged by NMORB-normalised bulk-rock Ce/Yb >1; NMORB of *37*). Shown for comparison are ratios for peridotitic cpx/garnet pairs (Woodland, unpubl. database) and for eclogitic cpx/garnet pairs produced in experiments[13]. (**B)** As in A. but distinguishing samples with low jadeite content (Jd) in cpx. Results for a regression through ten high-Jd samples plus one low-Jd sample are shown. (**C**) Calculated $Fe^{3+}/\Sigma Fe$ in cpx (calculated from garnet $Fe^{3+}/\Sigma Fe$ and temperature using the regression in (B) vs. measured $Fe^{3+}/\Sigma Fe$ in cpx. Resultant uncertainties are large due to the large uncertainties in the regression. (**D)** Calculated $Fe^{3+}/\Sigma Fe$ in the whole rock (using calculated $Fe^{3+}/\Sigma Fe$ in the cpx and measured $Fe^{3+}/\Sigma Fe$ in the garnet) vs. $Fe^{3+}/\Sigma Fe$ in the whole rock reconstructed from measured $Fe^{3+}/\Sigma Fe$ in cpx and garnet, assuming a proportion of 0.45 and 0.55, respectively. Uncertainties are propagated as described in Methods.

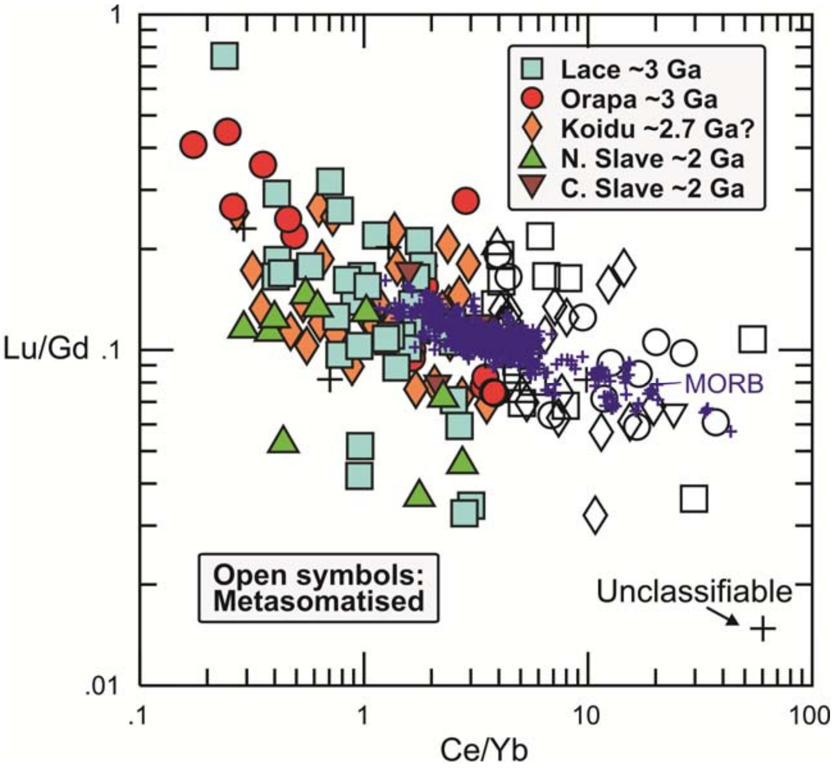

Supplementary Figure 7. REE systematics in mantle eclogites and MORB[48]. Open symbols distinguish samples that have been metasomatised, as gauged by NMORB-normalised bulk-rock Ce/Yb >1 (NMORB of *37*). Filled symbols are unmetasomatised samples the Ce/Yb of which decreases and Lu/Gd increases as a function of melt loss from eclogite, in warm Palaeoproterozoic and Archaean subduction zones[14]. Eclogite values extend far beyond the range of MORB, suggesting that normalisation to NMORB to gauge melt extraction is a valid approach. Conversely, several MORB samples extend to high Ce/Yb, suggesting that similar ratios in some mantle eclogites may be inherited from the ocean floor, and that they could be misidentified as metasomatised.

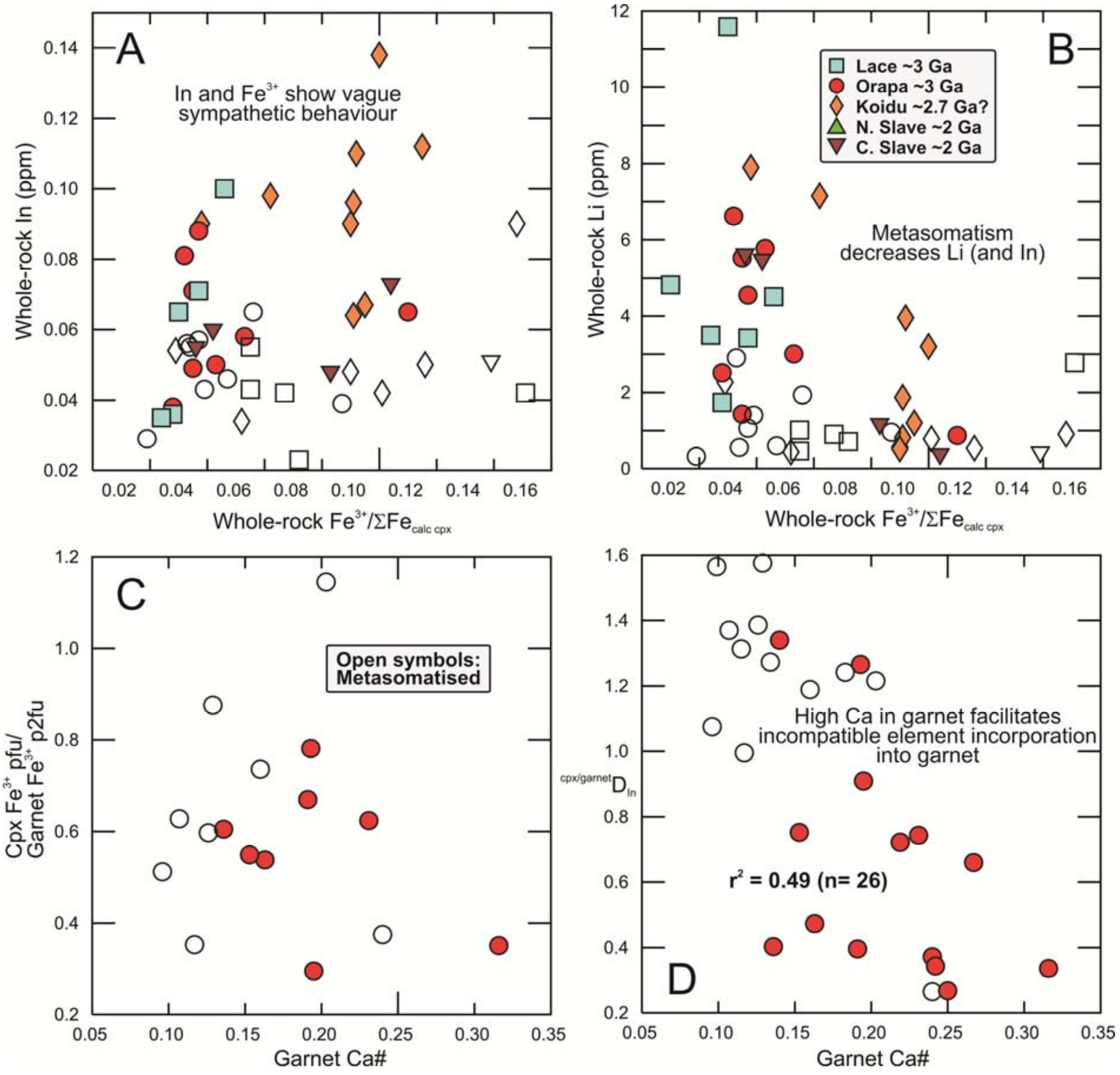

Supplementary Figure 8. $Fe^{3+}/\Sigma Fe$ and elemental relationships in mantle eclogites. (**A**) Indium and (**B**) Li as a function of $Fe^{3+}/\Sigma Fe$ in reconstructed whole rocks. Metasomatism, as gauged by NMORB-normalised bulk-rock Ce/Yb >1 (NMORB of *37*), appears to lead to lower In and Li (compare open to filled symbols); there is some correspondence between In and $Fe^{3+}/\Sigma Fe$ in unmetasomatised samples, as expected given their similar partitioning behaviour in MORB[6]. Lithium may be additionally affected by seawater alteration. The distribution of Fe^{3+} per formula unit (pfu) between cpx and garnet shows no clear relationship with garnet Ca# (**C**) although a negative correlation is observed for In (**D**), consistent with increased partitioning of incompatible trace elements into garnet with increasing CaO (e.g.[47]).

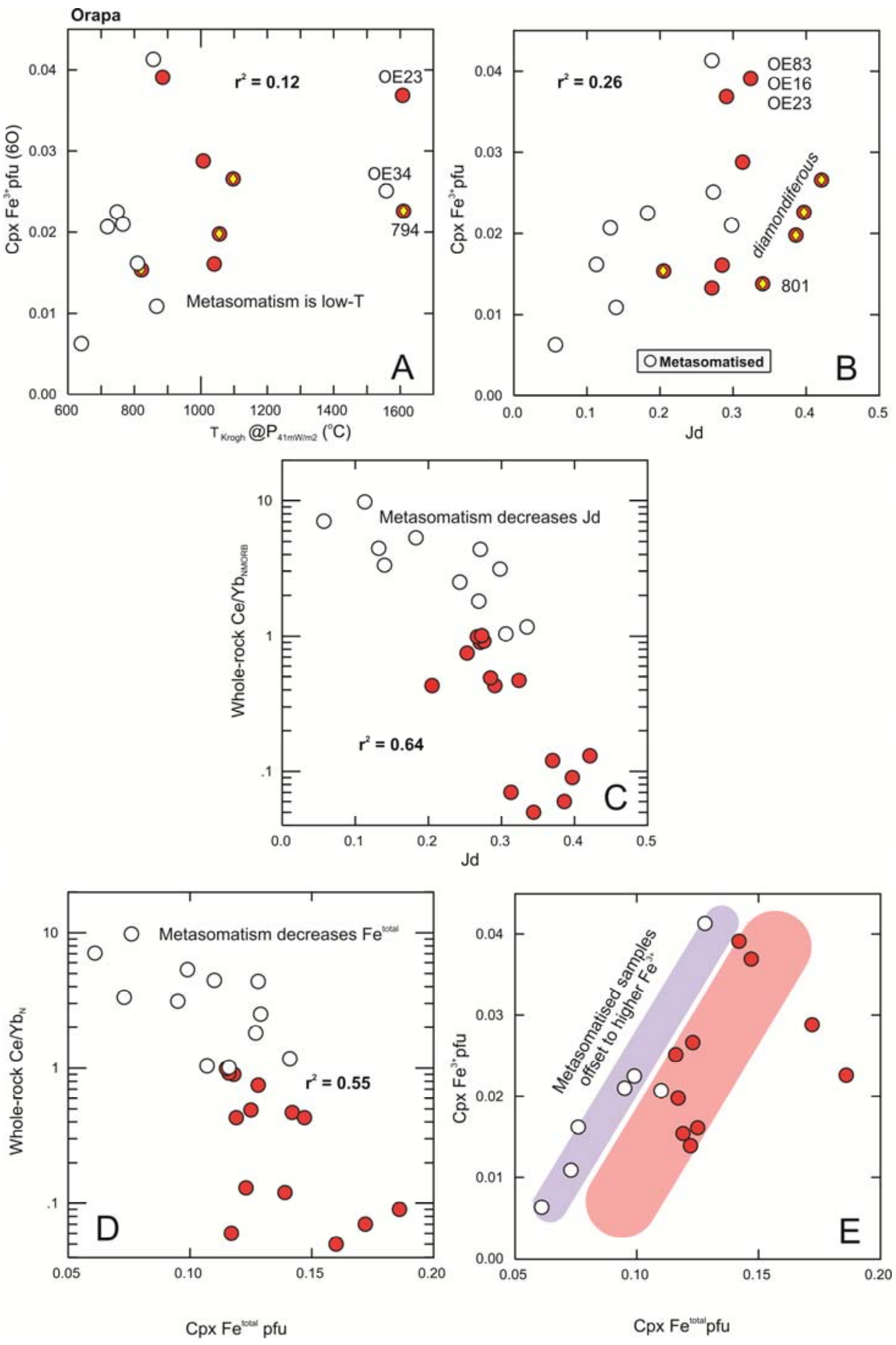

Supplementary Figure 9. Effects of crystal chemistry and metasomatism on $Fe^{3+}/\Sigma Fe$ in cpx. Fe^{3+} in cpx per formula unit (pfu, based on 6 oxygens) as a function of (**A**) temperature (derived as described in Supplementary Text) and (**B**) jadeite content in cpx (Jd). Open symbols show metasomatised samples (NMORB-normalised bulk-rock Ce/Yb>1; denoted by subscript NMORB; NMORB of *37*); small yellow diamonds distinguish diamondiferous samples, all non-metasomatised. The highest Fe^{3+} pfu are recorded in higher-temperature samples with higher Jd, which also tend to be non-metasomatised. This is illustrated in (**C**), which shows calculated whole-rock Ce/Yb_N as a function of Jd. However, metasomatism also leads to a decrease in total Fe pfu in cpx, as shown in (**D**). Thus, at a given total Fe content, metasomatised samples have higher Fe^{3+} pfu than non-metasomatised ones (**E**).

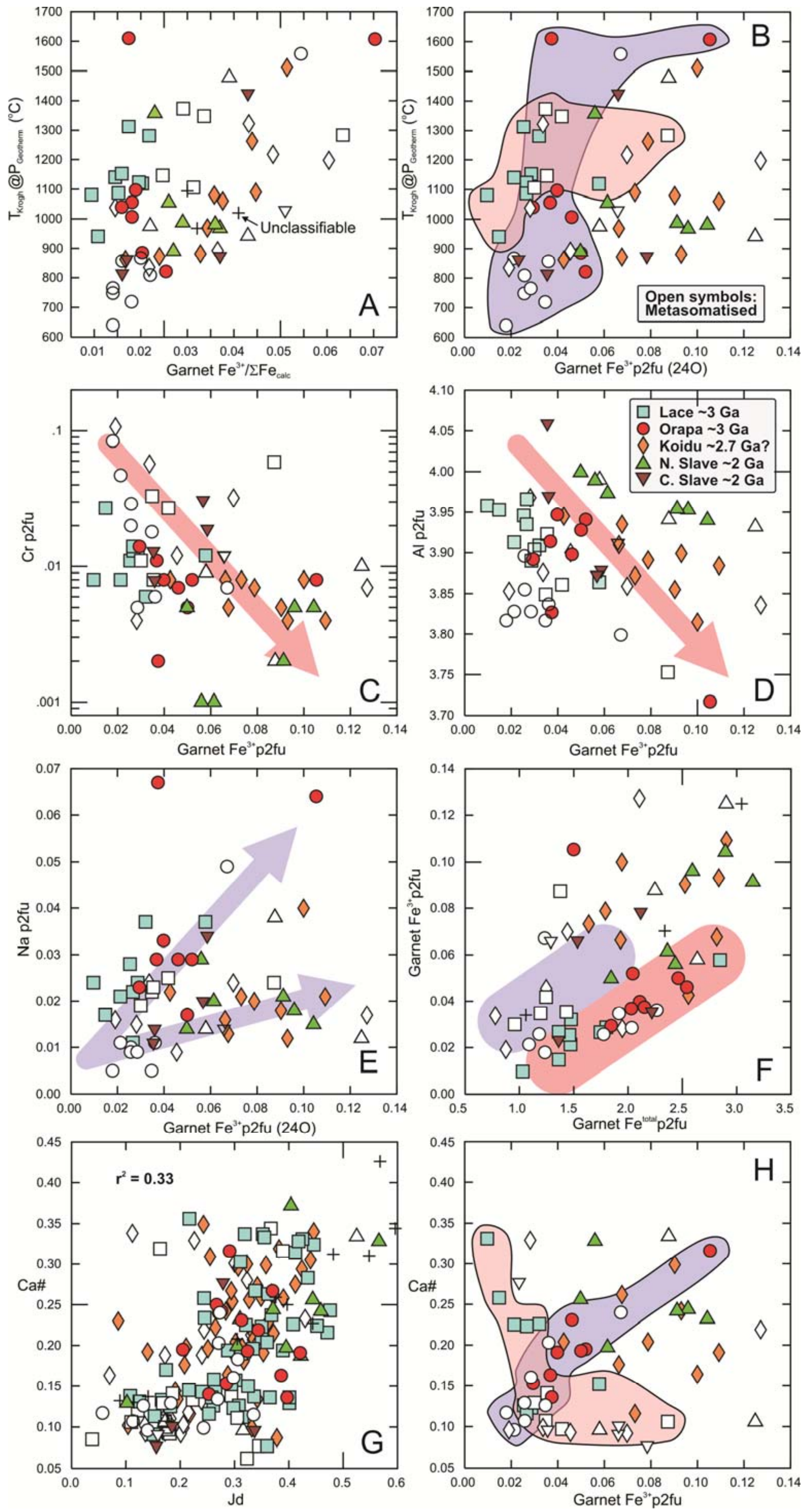
A
B
C
D
E
F
G
H
Unclassifiable
Open symbols: Metasomatised
Lace ~3 Ga
Orapa ~3 Ga
Koidu ~2.7 Ga?
N. Slave ~2 Ga
C. Slave ~2 Ga
r² = 0.33
T Krogh @P Geotherm (°C)
Garnet Fe3+/ΣFe calc
Garnet Fe3+ p2fu (24O)
Cr p2fu
Al p2fu
Garnet Fe3+ p2fu
Na p2fu
Garnet Fe total p2fu
Ca#
Jd

Supplementary Figure 10. Crystal-chemical controls on $Fe^{3+}/\Sigma Fe$ in garnet. (**A)** Garnet $Fe^{3+}/\Sigma Fe$ and (**B**) Fe^{3+} in garnet per two formula units (p2fu, based on 24 oxygens) vs. temperature (derived as described in Supplementary Text). Open symbols distinguish samples that have been metasomatised, as gauged by NMORB-normalised bulk-rock Ce/Yb >1 (NMORB of *37*). The various sample suites show variably strong positive correlations in (A) whereas this relationship is deteriorated for most sample suites when cations pfu are considered (B). Still, the highest Fe^{3+} in garnet appear to be recorded at high temperatures for eclogite suites from Lace and Orapa, whereas the other suites do not show coherent behaviour. Fe^{3+} in garnet p2fu as a function of (**C**) Cr, (**D**) Al and (**E**) Al p2fu. There appears to be a vague positive trend with monovalent Na, enabling coupled substitution with Fe^{3+} for two divalent cations[12,44,45], whereas competitive behaviour with other trivalent cations would be evident in negative trends with Cr and Al, which are not clearly observed. (**F**) Fe^{3+} p2fu as a function of total Fe p2fu in garnet. The highest Fe^{3+} at a given total Fe content appear to be recorded for metasomatised samples from Lace and the northern Slave craton, whereas other eclogite suites do not show such systematics. **(G)** illustrates that samples with high Ca# ($Ca/(Ca+Fe^{total}+Mg+Mn)$ molar) in garnet also tend to have high jadeite content (Jd) in coexisting cpx. In **(H)** contrasting behaviour with respect to Ca#-Fe^{3+} p2fu relationships in garnet is evident for eclogites from Lace (negative trend) and Orapa (positive trend), whereas the other eclogite suites show no relationship. This may be due to competing effects of temperature and crystal chemistry, where compositions reflect igneous differentiation of the magmatic protolith and metasomatism after emplacement in cratonic lithosphere.
